# Oncogenic circTICRR suppresses autophagy via binding to HuR protein and stabilizing GLUD1 mRNA in cervical cancer

**DOI:** 10.1038/s41419-022-04943-1

**Published:** 2022-05-20

**Authors:** Tingjia Zhu, Yixuan Cen, Zhuoye Chen, Yanan Zhang, Lu Zhao, Jiaying Wang, Weiguo Lu, Xing Xie, Xinyu Wang

**Affiliations:** 1 Women’s Reproductive Health Laboratory of Zhejiang Province, Women’s Hospital, School of Medicine, Zhejiang University, Hangzhou, 310006 Zhejiang China; 2 Department of Gynecologic Oncology, Women’s Hospital, School of Medicine, Zhejiang University, Hangzhou, 310006 Zhejiang China; 3 Department of Obstetrics and Gynecology, The Seventh Medical Centre, Chinese PLA General Hospital, Beijing, China; 4 Cancer center, Zhejiang University, Hangzhou, 310058 Zhejiang China; 5 Department of Gynecology and Obstetrics, The First Affiliated Hospital School of Medicine, Zhejiang University, Hangzhou, 310003 Zhejiang China

**Keywords:** Macroautophagy, Oncogenes

## Abstract

Circular RNAs (circRNAs) are critical regulators in the occurrence and development of numerous cancers, in which abnormal autophagy plays a key role. However, the potential involvement of circRNAs in autophagy is largely unknown. Here, we identified the overexpression of circTICRR, a circular RNA, in cervical cancer. In vitro experiments showed that knockdown of circTICRR activated autophagy, and consequently promoted apoptosis and inhibited proliferation in cervical cancer cells, and vice versa. CircTICRR interacted with HuR protein via binding to F287/F289 in the RRM3 domain of HuR, stabilizing GLUD1 mRNA and elevating the level of GLUD1 protein. In vivo experiments revealed that knockdown of circTICRR suppressed the growth of transplanted tumors. An inhibitory peptide specific to the binding site between circTICRR and HuR protein promoted autophagy, induced apoptosis, suppressed proliferation in cervical cancer cells, and inhibited the growth of xenografts. Our findings suggest that circTICRR acts as an oncogene in cervical cancer and the interaction between circTICRR and HuR protein may be a potential target in cervical cancer therapeutics.

## Introduction

Cervical cancer is the fourth most common and fatal malignancy among women worldwide [1], with an estimated 604 127 new cases and 341 831 deaths in 2020 (https://seer.cancer.gov/). There is still a high proportion of advanced cervical cancer at initial diagnosis, accounting for 52% of cases with regional involvement or distant metastasis, due to limited vaccination and screening in undeveloped countries or regions (SEER database). Patients with advanced cervical cancer usually lose the opportunity for surgery and present the unsatisfied response to radiotherapy and chemotherapy, and the 5-year relative survival of patients with distant metastasis is only 17.1% (SEER database). Different targeted therapies, including anti-angiogenic agents [2–4] and immune checkpoint inhibitors [5, 6], are demonstrating encouraging prospects for persistent, recurrent, and metastatic diseases, but they do not yet meet clinical requirements [7]. Hence, finding new molecular targets is urgent for cervical cancer therapeutics.

Circular RNAs (CircRNAs) are covalently closed structures without a 5’ cap and 3’ ployadenlated tail [8, 9]. Emerging evidences have revealed that circRNAs are involved in the occurrence and development of various cancers, including cervical cancer [9]. CircRNAs function as competing endogenous RNAs (ceRNAs) by sponging miRNAs, and consequently regulating the downstream target genes expression [10]. Recently, it was also found that circRNAs could bind to RNA binding proteins (RBPs) [11, 12] and even encoded peptides to play biological roles [13]. For example, circAGO2 drives gastric cancer progression through binding with HuR [11], and circβ-catenin produces a 370-amino acid β-catenin isoform to promote liver cancer cell growth [13]. Although the expression of circRNAs is tissue-specific, previous studies have provided a reference to explore new therapeutic targets for cervical cancer.

Cell death is the final effect of all drugs. The types of cell death include: necrosis, apoptosis, autophagy, ferroptosis, and others [14–16]. Among them, autophagy is a programmed cell death pathway, and its dysfunction is not only related to tumorigenesis, but also associated with the drug sensitivity of cancer cells [17, 18]. It has been shown that activation of autophagy is necessary to effectively kill cancer cells [19], which is called autophagic cell death [20]. A recent study found that silencing of circHIPK3 induced autophagy, via MIR124-3p-STAT3-PRKAA/AMPKa axis, to suppress tumor growth in lung cancer [21], suggesting that circRNAs participate in the regulation of autophagy. Hence, circRNAs, which possess the ability to regulate autophagy, are expected to have the potential of the therapeutic target of cancers.

In a previous study of ours, we identified an up-regulated circRNA, circTICRR, in cervical cancer tissues, but the function of circTICRR is unknown up to date. In this study, we found that circTICRR promoted cervical cancer cells proliferation and inhibited apoptosis in vitro and in vivo through restraining autophagy, and that circTICRR functioned via directly interacting with HuR protein to stabilize GLUD1 mRNA. GLUD1 has been proved to be a mitochondrial matrix enzyme in glutaminolysis and contributes to the control of autophagy to regulate the malignant phenotype of cancer cells [22–25]. We further confirmed that circTICRR knockdown or application of a specific peptide to reduce or block the interaction between circTICRR and HuR activated autophagy, induced cell death, and inhibited the growth of subcutaneously transplanted tumors. Our study aimed to demonstrate an oncogenic role and underlying mechanism of circTICRR, so as to provide the evidences for the potential of circTICRR as a target in cervical cancer therapeutics.

## Results

### Identification and characterization of circTICRR in cervical cancer

**Fig. 1:**
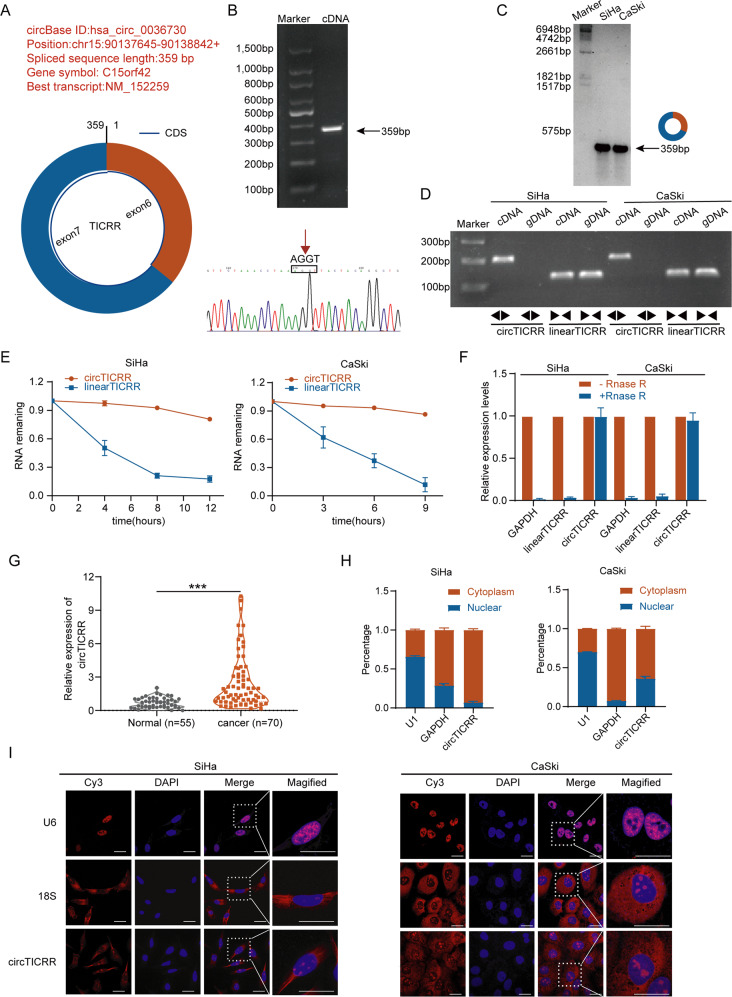
Identification and characterization of circTICRR in cervical cancer. **A** The exonic information of circTICRR (circBase ID: hsa_circ_0036730) is illustrated as indicated. **B** The qRT-PCR product using full length primers (up) and back-splice junction sequences (down) of circTICRR was determined by agarose gel electrophoresis and Sanger sequencing, respectively. **C** Northern blot using a junction-specific DIG-labeled probe showed the endogenous existence of circTICRR in SiHa and CaSki cells. **D** PCR and agarose gel electrophoresis assays detected circTICRR using divergent and convergent primers from complementary DNA (cDNA) or genomic DNA (gDNA) in SiHa and CaSki cells. Linear TICRR was used as an mRNA control. **E** The qRT-PCR detected the abundance of circTICRR and linear TICRR in the SiHa and CaSki cells treated with actinomycin D (5 μg/ml) at the indicated time points. **F** The relative RNA expression levels were examined by qRT-PCR after treatment with RNase R or mock in total RNAs extracted from SiHa and CaSki cells. **G**.The relative expression level of circTICRR was detected by qRT-PCR in 55 normal cervical and 70 cervical cancer tissues. 18 s was used as a loading control. **H** The distribution of circTICRR was detected by qRT-PCR in SiHa and CaSki cells. U1 and GAPDH were used as nuclear and cytoplasmic positive controls, respectively. **I** The cellular distribution of circTICRR was detected by FISH analysis. Red indicates circTICRR with a Cy3-labeled probe. The nuclei were stained with DAPI (blue). Scale bar, 20 μM. Data are representative of at least three independent experiments and presented as the mean ± SD. *p < 0.05, **p < 0.01, ***p < 0.001.

Previously, we had discovered some differentially expressed circRNAs in 6 human cervical cancer and 6 normal tissues through Ribo-zero RNA sequencing (available in GEO database: GSE147009) [26]. Among them, hsa_circ_0036730 (termed as circTICRR) was found to be up-regulated in cervical cancer tissues (fold change = 4.2, *p* < 0.05) and was validated to be a circRNA in circBase (Fig. 1A). Thus, circTICRR was chosen for further study. The RT-PCR product amplified by full-length primers designed for circTICRR revealed that circTICRR is a 359-nucleotide (nt) circRNA, which was consistent with circBase (Fig. 1B up), and the sequence of the products and the back-spliced junction were confirmed by Sanger sequencing (Fig. 1B down). Besides, endogenous expression of circTICRR in the SiHa and CaSki cells, two human squamous cell carcinoma cell lines, was detected using a junction-specific probe in Northern Blot assay (Fig. 1C). In addition, the cyclization and stability of 359-nt circTICRR were validated by divergent primers, actinomycin-D, and Ribonuclease R treatment (Fig. 1D-F).

We further evaluated the levels of circTICRR expression in 55 cervical normal and 70 cancer tissues. CircTICRR expression was observed to be significantly up-regulated in cervical cancer tissues compared with in cervical normal tissues, which was consistent with the RNA-seq results (Fig. 1G).

Cytosolic/nuclear fractionation and RNA fluorescence in situ hybridization (FISH) assays showed that circTICRR was mainly located in the cytoplasm (Fig. 1H, I).

### CircTICRR plays an oncogenic role in cervical cancer in vitro and in vivo

**Fig. 2:**
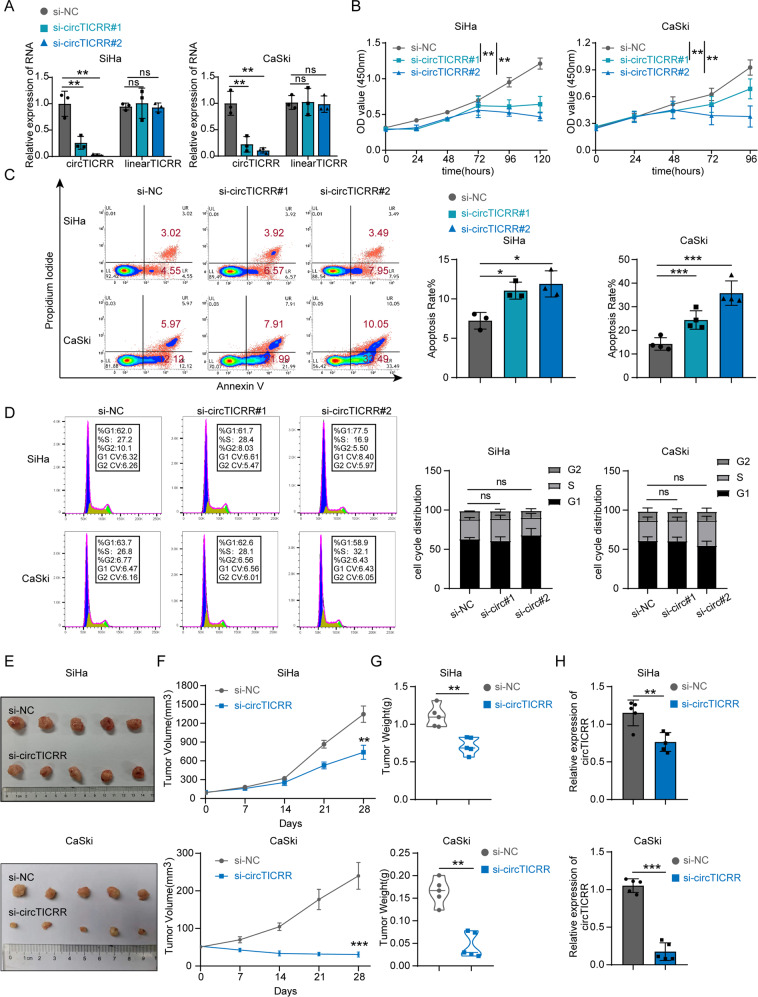
CircTICRR plays an oncogenic role in cervical cancer in vitro and in vivo. **A** The relative expression levels of circTICRR or TICRR mRNA were detected by qRT-PCR in the SiHa and CaSki cells transfected with two junction-specific siRNAs targeting circTICRR or corresponding negative control. Cell viability (**B**) was determined by CCK-8 assays, cell apoptosis (**C**), and cell cycle (**D**) was measured by Flow Cytometry in the SiHa and CaSki cells transfected with two circTICRR back splicing-specific siRNAs or a negative control siRNA. Images of tumors **E**, tumor volume (**F**) and weight (**G**), and the relative levels of circTICRR expression (**H**) in the xenografts treated with si-circTICRR#2 or negative control siRNA after removing the tumors (*n* = 5 for each group). Data are representative of at least three independent experiments and p*resented as the mean ± SD. *p < 0.05, **p < 0.01, ***p < 0.001.

To explore the biological function of circTICRR in cervical cancer cells, two junction-specific siRNAs of circTICRR were transfected into SiHa and CaSki cells, and circTICRR expression was verified to be significantly down-regulated in both cell lines, but linear TICRR mRNA expression did not change (Fig. 2A). The CCK-8 assay revealed that circTICRR knockdown notably inhibited cell proliferation (Fig. 2B), and flow cytometry demonstrated that circTICRR knockdown promoted cellular apoptosis, but did not alter the cell cycle, in SiHa and CaSki cells (Fig. 2C, D).

We also constructed a plasmid to overexpress circTICRR in SiHa and CaSki cells, but failed (Fig. S1a). Thus, we detected the basal expression levels of circTICRR in different cervical cell lines, and found the lowest level of circTICRR in HeLa cells (Fig. S1b). Therefore, we transfected the plasmid into HeLa cells and found that circTICRR expression was successfully up-regulated (Fig. S1c,d). As expected, the CCK-8 assay and flow cytometry showed that stable overexpression of circTICRR promoted cell proliferation but inhibited apoptosis of HeLa cells (Fig. S1e,f).

To investigate the effect of circTICRR on cervical cancer in vivo, we established subcutaneous transplanted tumor models in nude mice using SiHa and CaSki cells. Using intratumoral injection of circTICRR siRNA#2 or negative control siRNA, we found that the growth, volume, and weight of xenografts treated with circTICRR siRNA were significantly inhibited (Fig. 2E-H). Hematoxylin and eosin (H&E) staining of xenografts tissues showed typical morphological character of cancer, and terminal deoxynucleotidyl transferase dUTP nick-end Labeling (TUNEL) analysis showed significantly increased apoptotic cells in the xenografts with circTICRR knockdown (Fig. S1g). Together, our results suggest that circTICRR plays an oncogenic role in cervical cancer in vitro and in vivo.

### CircTICRR knockdown promotes apoptosis via inducing autophagy in cervical cancer cells

**Fig. 3:**
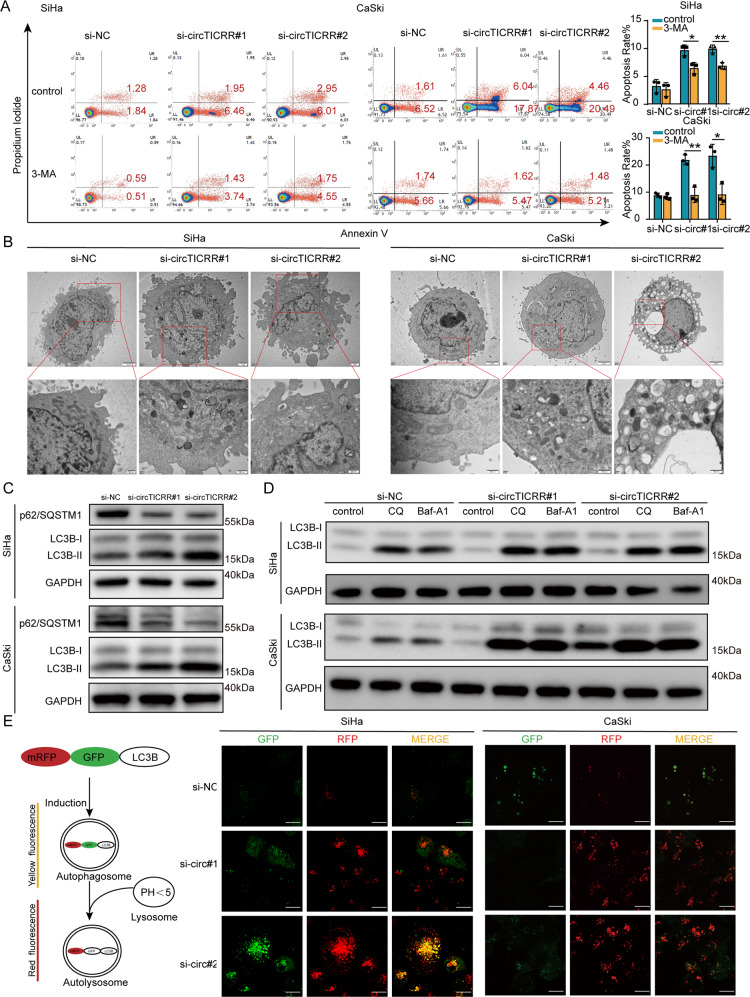
CircTICRR knockdown promotes apoptosis via inducing autophagy in cervical cancer cells. **A** Apoptosis in SiHa and CaSki cells was analyzed by Flow Cytometry. Cells were treated with autophagy inhibitor (3-MA) or normal medium for 1 h, then combined treated with siRNAs for additional 72 h. Cell morphology (**B**) and autophagy-related proteins (**C**) were monitored by TEM and western blot, respectively. **D** SiHa and CaSki cells transfected with siRNAs or negative control for 72 h were treated with the autophagy inhibitor CQ or Baf-A1 for another 6 h. Then, the autophagy-related protein LC3BI/II was monitored by western blotting. **E** Left panel: Schematic diagram illustrating the mechanism of mRFP-GFP-LC3B. Right panel: SiHa and CaSki cells transfected with mRFP-GFP-LC3B for 24 h were transfected with si-NC, si-circTICRR#1 and #2, respectively, for another 48 h. Images were captured by confocal microscopy. Scale bar, 30 μm. Data are representative of at least three independent experiments and presented as the mean ± SD. *p < 0.05, **p < 0.01, ***p < 0.001.

To further investigate the mechanism by which circTICRR promotes apoptosis in cervical cancer cells, we treated SiHa and CaSki cells with two siRNAs specific to circTICRR and different cell death inhibitors, including the autophagy inhibitor 3-methyladenine (3-MA), the caspase/apoptosis inhibitor Z-VAD-FMK, the Necrooptosis inhibitor Necrostatins 1, the pyroptosis inhibitor Ferrostatin-1, and liproxstatin-1, and found that only 3-MA partially reversed apoptosis induced by circTICRR knockdown in SiHa and CaSki cells (Fig. 3A, Fig. S2a), suggesting that circTICRR knockdown promotes apoptosis probably via inducing autophagy in cervical cancer cells.

Therefore, we evaluated the effect of circTICRR on autophagy in cervical cancer cells. We observed increased autophagosomes (Fig. 3B), significantly up-regulated LC3B-II expression levels, increased LC3B-I/II conversion, and p62/SQSTM1 degradation in the SiHa and CaSki cells with circTICRR knockdown (Fig. 3C), and the opposite results were obtained in HeLa cells with overexpressed circTICRR (Fig. S2b,c), compared with the control cells, suggesting that circTICRR knockdown increased the amount of autophagosomes in cervical cancer cells, and vice versa.

We further monitored the change of autophagic flux caused by circTICRR knockdown. We utilized two autophagy flux inhibitors, chloroquine (CQ) and bafilomycin A1 (Baf-A1), to treat the SiHa and CaSki cells with circTICRR knockdown. In the presence of CQ or Baf-A1, the LC3B-II levels in the cells with down-regulated circTICRR were still increased (Fig. 3D). Furthermore, we detected the autophagic flux using the Hanbio mRFP-GFP-LC3B adenovirus. Both mRFP and GFP are fluorescence emitting proteins fused with LC3B which is expressed in autophagosomes, while fusion with lysosomes to form autolysosomes, the GFP signal is quenching owing to the low pH environment, remaining only mRFP signal. More Red signal levels were observed in the SiHa and CaSki cells with circTICRR knockdown (Fig. 3E). Together, our results suggest that circTICRR knockdown promotes the formation of total autophagosomes by accelerating autophagic flux in cervical cancer cells.

### GLUD1 participates in circTICRR modulating the viability and autophagy of cervical cancer cells

**Fig. 4:**
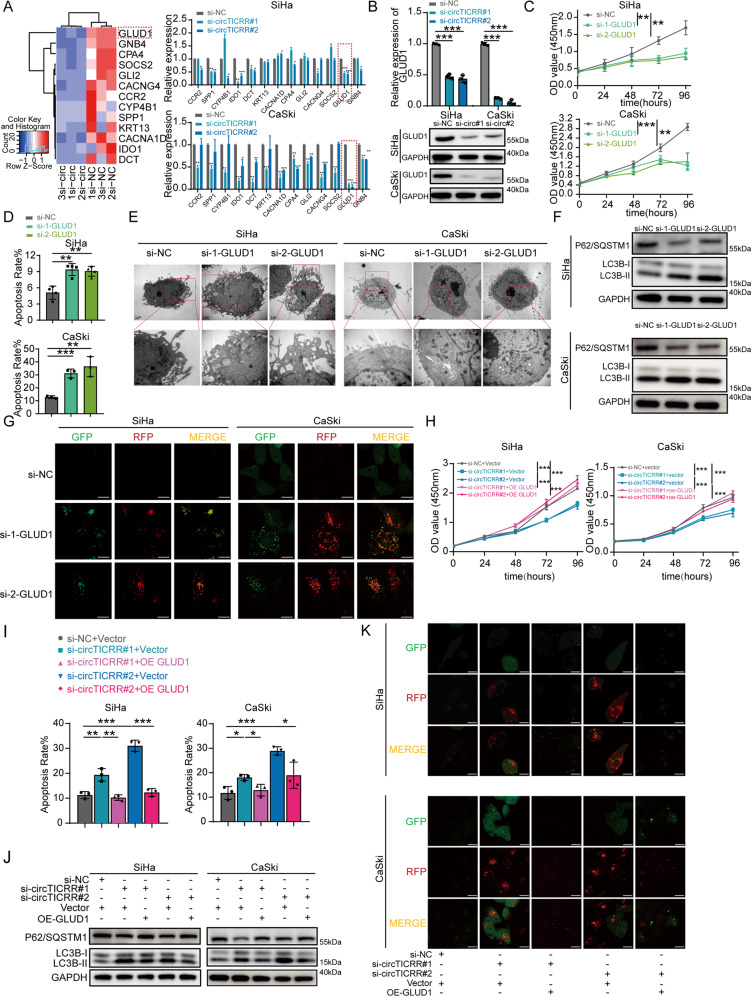
GLUD1 participates in circTICRR modulating the viability and autophagy of cervical cancer cells. **A** Left panel: Part of the cluster heatmap of differentially expressed mRNAs in the SiHa cells transfected with si-circTICRR#2 or negative control by RNA-seq. Each group had three repeats. Right panel: qRT-PCR analysis of 13 chosen top differentially expressed mRNAs in the SiHa and CaSki cells treated with two siRNAs targeting circTICRR or si-NC. **B** The relative GLUD1 mRNA (up) and protein (down) levels in the SiHa and CaSki cells transfected with two circTICRR siRNAs or negative control. Cell viability (**C**) was determined by CCK-8 assay, cell apoptosis (**D**) was measured by Flow Cytometry, cell morphology (**E**) was monitored by TEM, and autophagy-related proteins (**F**) were detected by western blot in the SiHa and CaSki cells transfected with two GLUD1 siRNAs or a negative control siRNA. **G** SiHa and CaSki cells transfected with mRFP-GFP-LC3B for 24 h were transfected with si-NC, si-circGLUD1#1 or #2 for another 48 h. Images were captured by confocal microscopy. Scale bar, 30 μm. SiHa and CaSki cells were transfected with si-circTICRR#1, si-circTICRR#2, si-circTICRR#1 plus GLUD1 plasmid, si-circTICRR#2 plus GLUD1 plasmid, and negative controls, respectively. CCK-8 assay (**H**) and Flow Cytometry (**I**) were performed to test cell viability. Autophagy-related proteins were detected by western blot **J**. Autophagy flux was monitored by confocal microscopy **K**. Scale bar, 30 μM. Data are representative of at least three independent experiments and presented as the mean ± SD. *p < 0.05, **p < 0.01, ***p < 0.001.

To identify the downstream target that participates in circTICRR modulating the viability and autophagy in cervical cancer cells, we conducted RNA sequencing (RNA-seq) in the SiHa cells transfected with or without si-circTICRR#2, and found a total of 2776 differentially expressed mRNAs (fold change>1.5, *P* < 0.05). Considering the significance of difference, stability, and function, we chose the 13 top differentially expressed mRNAs to validate their expression levels. Among those candidates, GLUD1 mRNA expression was most significantly down-regulated, as well as GLUD1 protein (Fig. 4A, B), in the SiHa cells with circTICRR knockdown, which was conversely validated between the HeLa cells with and without circTICRR overexpression (Fig. S3a).

We further investigated the effect of GLUD1 on the viability and autophagy of cervical cancer cells. Our results revealed that GLUD1 knockdown, using two siRNAs and a potent inhibitor of glutamate dehydrogenase 1, inhibited cell proliferation and promoted apoptosis (Fig. 4C, D and Fig. S3b-e), and GLUD1 knockdown enhanced autophagosomes and autolysosomes (Fig. 4E–G and Fig. S3f). Furthermore, the overexpression of GLUD1 significantly revised inhibited cell viability and enhanced autophagy induced by circTICRR knockdown (Fig. 4H–K). Conversely, GLUD1 knockdown reversed the enhanced cell viability and inhibited autophagy induced by circTICRR overexpression in HeLa cells (Fig. S3h-k).

Together, our results suggest that GLUD1 participates in circTICRR modulating the viability and autophagy of cervical cancer cells

### CircTICRR interacting with HuR stabilizes GLUD1 mRNA

**Fig. 5:**
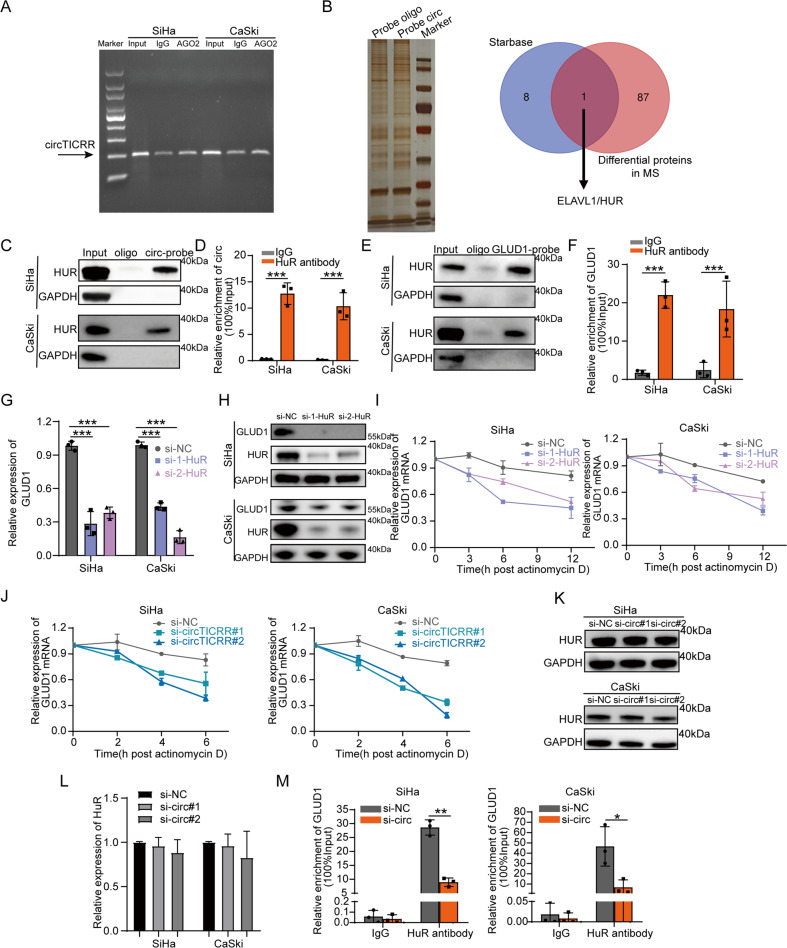
CircTICRR interacting with HuR stabilizes GLUD1 mRNA. **A** The binding of circTICRR to the AGO2 protein was determined by agarose gel electrophoresis assay. **B** Left panel: RNA pull-down assay of SiHa cells using a junction-specific circTICRR probe or an oligo probe. Sliver staining and MS then were performed to analyze the pulled down differentially expressed proteins. Right panel: Venn diagram showing the overlap results of the differentially expressed proteins in MS and circTICRR targets in StarBase. **C, D**. Protein pulled down by control oligo or circTICRR probe with HuR antibody was detected by western blot (**C**), and enriched circTICRR using HuR antibody was detected by qRT-PCR after RIP assay (**D**) of SiHa and CaSki cells. Data were calculated as input %. **E, F**. Protein pulled down by control oligo or GLUD1 mRNA probe with HuR antibody was detected by western blot (**E**) and enriched GLUD1 mRNA using HuR antibody was detected by qRT-PCR after RIP assay (**F**) of SiHa and CaSki cells. Data were calculated as input %. The relative levels of GLUD1 mRNA (**G**) and protein **H** expression in the SiHa and CaSki cells with HuR knockdown were detected by qRT-PCR and western blot, respectively. **I** SiHa and CaSki cells transfected with a negative control siRNA or two HuR siRNAs were treated with actinomycin D (5 μg/ml) at the indicated time points. The relative level of GLUD1 mRNA expression was detected by qRT-PCR. **J** SiHa and CaSki cells transfected with a negative control siRNA or two circTICRR siRNAs were treated with actinomycin D (5 μg/ml) at the indicated time points. The relative GLUD1 mRNA level was detected by qRT-PCR. The relative level of HuR expression in the SiHa and CaSki cells with circTICRR knockdown was detected by western blot (**K**) and qRT-PCR **L**, respectively. **M**. GLUD1 mRNA recruited by HuR antibody from the lysates in SiHa cells with or without circTICRR knockdown was detected by RIP assay followed by qRT-PCR. Data are representative of at least three independent experiments and presented as the mean ± SD. *p < 0.05, **p < 0.01, ***p < 0.001.

To further explore how circTICRR regulates the expression of GLUD1, we initially guessed that mainly cytoplasm-localized circTICRR might function as a miRNA sponge similar to other ncRNAs [8]. However, the result did not show that circTICRR bound to AGO2 (Fig. 5A).

Then, we performed mass spectrometry (MS) analysis after RNA pull-down to explore the protein binding role of circTICRR. Interestingly, only RBP-HuR was overlapped in both MS and circTICRR CLIP-seq dataset derived from starbase (Fig. 5B). RNA pull-down and RNA Binding Protein Immunoprecipitation (RIP) assay identified the interaction between circTICRR and HuR in SiHa and CaSki cells (Fig. 5C, D). Simultaneously, we confirmed the interaction between HuR and GLUD1 mRNA (Fig. 5E, F), implying that HuR probably played a role in the interaction between circTICRR and GLUD1 mRNA during circTICRR regulation of GLUD1 expression.

As HuR is crucial for mRNA stability [27] and plays an oncogenic role in cancers [28, 29], and GLUD1 mRNA and protein levels were down-regulated by HuR knockdown in this study (Fig. 5G, H), we further explored the stability of GLUD1 mRNA by HuR knockdown followed by ACTD treatment, and found that the stability of GLUD1 mRNA was decreased in the SiHa and CaSki cells with HuR knockdown, compared with control cells (Fig. 5I).

We further found that circTICRR knockdown also decreased the GLUD1 mRNA stability (Fig. 5J). Because circTICRR directly binds to HuR protein and circTICRR knockdown did not alter the level of HuR mRNA and protein expression (Fig. 5K, I), we performed RIP assay and found a decreased percentage of GLUD1 mRNA recruited to HuR in the SiHa and CaSki cells with circTICRR knockdown (Fig. 5M). Together, our results suggest that circTICRR interacts with the HuR protein, subsequently enhancing the stability of GLUD1 mRNA in cervical cancer cells.

### HuR Inhibitory peptide suppresses cervical cancer progression in vitro and in vivo by blocking circTICRR-HuR interaction

**Fig. 6:**
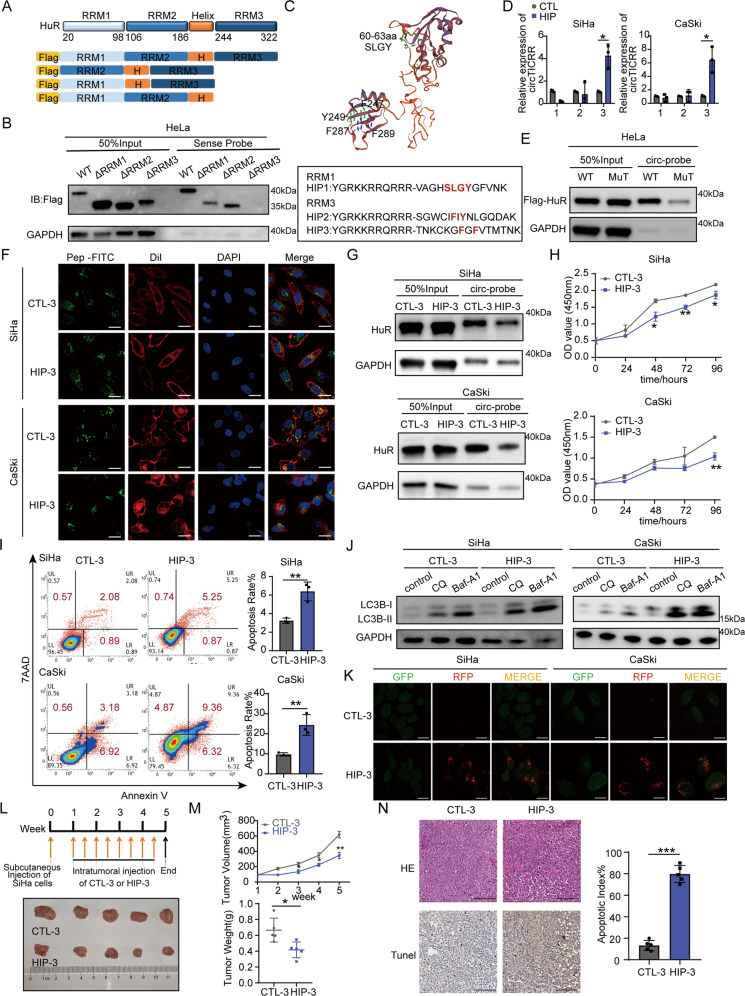
HuR Inhibitory peptide suppresses cervical cancer progression in vitro and in vivo by blocking circTICRR-HuR interaction. **A** The schematic domain structure of HuR. **B** In vitro RNA pull-down assay depicting the circTICRR-HuR interaction from HeLa cells validated by western blot after incubation with full-length, or truncated3XFlag-tagged recombinant HuR protein. **C** Upper panel: the schematic structure of the SLGY (60-63 aa), F247/Y249, and F287/F289 residues of the HuR protein crucial for interacting with circTICRR. Lower panel: the inhibitory peptides sequence containing the core amino acids of HuR. **D** Biotin-labeled peptide pull-down assay revealing the interaction of Control peptide (CTL) (30 μmol/L) or HIP (30 μmol/L) with circTICRR in HeLa cells. **E** In vitro RNA pull-down assay depicting the circTICRR/HuR interaction from HeLa cells validated by western blot after incubation with full-length, or mutant F287A/F289A of 3XFlag-tagged recombinant HuR protein. **F** Confocal images showing the distribution of synthesized inhibitory peptide (HIP-3: 30 μmol/L) or control peptide (CTL-3: 30 μmol/L) after incubation with SiHa and CaSki cells for 48 h. The nuclei were stained with DAPI (blue), while the membrane was stained with DiI (red). Scale bar, 20 μm. **G** Protein pulled down by control oligo or circTICRR probe with HuR antibody was detected by western blot. Cell viability (**H**) was determined by CCK-8 assay, cell apoptosis (**I**) was measured by Flow Cytometry in the SiHa and CaSki cells treated with HIP-3 or CTL-3. **J** SiHa and CaSki cells treated with HIP-3 or CTL-3 for 72 h were incubated with the autophagy inhibitor CQ or Baf-A1 for another 6 h. Then the autophagy-related protein LC3B I/II was monitored by Western blotting. **K** SiHa and CaSki cells transfected with mRFP-GFP-LC3B for 24 h were incubated with HIP-3 or CTL-3 for another 48 h. The images were captured by confocal microscopy. Scale bar, 20 μm. Images of tumors **L**, tumor volume and weight **M**, images of H&E and apoptotic cells (**N**) in the xenografts treated with HIP-3 or CTL-3 after collection of the tumors (*n* = 5 for each group). Scale bar, 100 μm. Data are representative of at least three independent experiments and presented as the mean ± SD. *p < 0.05, **p < 0.01, ***p < 0.001.

HuR is an RNA-binding protein including three RNA recognition motifs (RRMs). Therefore, we constructed 3xFlag-tagged HuR protein and three other HuR RRM domain-truncated plasmids (Fig. 6A). The abilities of HeLa cells transfected with these plasmids to bind to circTICRR were detected by Flag protein immunoblotting analysis after the RNA pull-down assay. We found that RRM3-truncated (244-322 amino acids [aa]) HuR almost completely and RRM1-truncated (20-98 aa) HuR partially lost the ability to bind to circTICRR, while RRM2-truncated (106-186 aa) HuR still retained such an ability, suggesting that RRM3, possibly RRM1 but not RRM2, was a crucial site for the interaction of HuR with circTICRR (Fig. 6B). To further confirm the exact sites in HuR interacting with circTICRR, we designed and synthesized cell-penetrating peptides, SLYG (60-63 aa), F247/Y249, and F287/F289, named HIP-1, HIP-2 and HIP-3, respectively, which had been recognized as critical residues for binding to RNAs in previous studies [11, 30, 31] (Fig. 6C). In vitro peptide pull-down assay showed that only HIP-3 could bind with endogenous circTICRR (Fig. 6D). Thus, we constructed F287A/F289A mutant plasmid and transfected it into HeLa cells. As expected, when full-length HuR plasmid was replaced with the F287A/F289A mutation, the interaction between circTICRR and HuR was diminished (Fig. 6E). The treatment with fluorescein isothiocyanate (FITC)-labeled HIP-3 or control peptide (CTL-3) resulted in obvious aggregation of peptide within the cytoplasm in SiHa and CaSki cells (Fig. 6F). What’s more, HIP-3 treatment reduced the endogenous circTICRR-HuR interaction (Fig. 6G), and consequently suppressed proliferation, promoted apoptosis, increased the formation of total autophagosomes, and accelerated autophagic flux in SiHa and CaSki cells (Fig. 6H-K). Consistent with in vitro experiment, intratumoral injection of HIP-3 significantly suppressed the growth of subcutaneous xenografts, including tumor volume and weight (Fig. 6L, M). H&E staining of xenografts tissues showed typical morphological character of cancer, and TUNEL analysis showed significantly increased cellular apoptosis in the xenografts after HIP-3 treatment (Fig. 6N). Together, our results suggest that HIP3, which specifically blocks the circTICRR-HuR interaction activates autophagy, promotes apoptosis, and consequently suppresses cervical cancer progression in vitro and in vivo.

## Discussion

In this study, we demonstrated, for the first time to our knowledge, an oncogenic role of circTICRR in cervical cancer. Our in vitro experiments showed that circTICRR knockdown inhibited proliferation and promoted apoptosis, but did not change the cell cycle, in cervical cancer cells. Furthermore, our in vivo experiments revealed that the growth of xenografts was inhibited when subcutaneously transplanted tumor cells were treated with circTICRR siRNA in nude mouse model. Importantly, we found that apoptosis was only reversed by autophagy inhibitor among different cell death inhibitors, suggesting that circTICRR suppressed apoptosis may be associated with inhibited autophagy. Previous studies have also shown that autophagy may take the lead in responding to stress stimuli and triggers cancer cell apoptosis, subsequently resulting in cell death [32, 33]. Thus, we performed a series of experiments associated with autophagy, and found that circTICRR knockdown increased autophagosomes, up-regulated LC3B-II expression levels, accelerated LC3B-I/II conversion, and degraded p62/SQSTM1 in cervical cancer cells. We further observed that the LC3B-II levels were still increased in the presence of the autophagy flux inhibitors, CQ and Baf-A1, in cells with circTICRR knockdown.

Furthermore, we monitored autophagy flux using mRFP-GFP-LC3B adenovirus. Thus, we found that circTICRR acts as an oncogene, via inhibiting autophagy, in cervical cancer.

To ascertain the signaling pathway by which circTICRR inhibits autophagy, we performed a RNA-seq and found significantly down-regulated expression of GLUD1 mRNA in the cervical cancer cells with circTICRR knockdown. Previous studies have showed that GLUD1 contributes to leucine sensing in the regulating of autophagy, affecting cell proliferation, metastasis, metabolism, and drug resistance in various cancers [22, 25, 34, 35], in which leucine acts as a potent suppressor of autophagy by activating MTORC1 that is an essential negative regulator of autophagy [36–40]. In this study, we observed that GLUD1 knockdown promoted autophagy, induced apoptosis, and inhibited cell proliferation, while overexpression of GLUD1 reversed autophagy activated by circTICRR knockdown, suggesting that GLUD1 is involved in circTICRR regulating autophagy in cervical cancer cells.

Among various mechanisms by which circRNA functions, miRNA sponge is mostly studied. The miRNA sponge refers to the change of downstream target gene expression caused by circRNA absorbing miRNA [41, 42]. Under such a phenomenon, circRNA, miRNA, and AGO2 often form a ternary complex [11], but we did not observe the interaction between circTICRR and AGO2 in this study, suggesting that there may be another intermediate molecule that participates in circTICRR regulating GLUD1 mRNA expression. Recent reports showed that RBP protein could also be bound to circRNAs in the regulation of the expression of downstream genes [12]. HuR, as a mediator of mRNA stability, is an RBP protein, which plays a vital role in the occurrence, development, and metastasis of cancers [29, 43]. As expected, we indeed observed that circTICRR could bind to HuR protein, knockdown of circTICRR reduced the binding of HuR to GLUD1, and knockdown of both circTICRR and HuR reduced the stability of GLUD1 mRNA, consequently decreasing the level of GLUD1 protein expression. Previous studies have shown that SLGY (60-63 aa) region of HuR RRM1 domain is a key site in the interaction with RNAs [11], and two residues (F247/Y249 and F287/F289) of RRM3 domain are the essential binding sites to form spatial structures affecting the RNA binding [30, 31]. We also found that the interaction between circTICRR and HuR protein was diminished when HuR plasmid with truncated RRM3 domain or F287/F289 mutation was used to replace full-length HuR plasmid. Together, our results suggest that the interaction between circTICRR and HuR, via F287/F289 in RRM3 domain of HuR, stabilizes GLUD1 mRNA and elevates the level of GLUD1 protein, leading to inhibition of autophagy in cervical cancer cells.

To explore the potential of circTICRR binding to HuR F287/F289 sites as a therapeutic target in clinic, we designed an inhibitory peptide specific to the binding site between circTICRR and HuR F287/F289, named HIP3, which could competitively block the binding between endogenous circTICRR and HuR protein. In vitro experiments showed that HIP3 activated autophagy, induced apoptosis, and inhibited proliferation in cervical cells, and in vivo experiments also demonstrated that HIP3 suppressed the growth of transplanted tumors in nude mouse model. Our findings suggest the potential value of HIP3, which can block the interaction between circTICRR and HuR protein, in cervical cancer therapeutics.

In summary, we found that circTICRR expression was upregulated in cervical cancer, and knockdown of circTICRR promoted apoptosis and inhibited proliferation, via activating autophagy, in cervical cancer cells, and vice versa. CircTICRR interacted with HuR protein via binding to F287/F289 in the RRM3 domain of HuR, stabilizing GLUD1 mRNA and elevating the level of GLUD1 protein. Knockdown of circTICRR suppressed the growth of transplanted tumors. An inhibitory peptide specific to binding the site between circTICRR and HuR protein promoted autophagy, induced apoptosis, and suppressed proliferation in cervical cells, and inhibited the growth of xenografts. Thus, we demonstrated an oncogenic role of circTICRR in cervical cancer and the underlying mechanism by which circTICRR inhibited autophagy, through the binding to HuR protein and stabilizing GLUD1 mRNA. Our findings suggest that the interaction between circTICRR and HuR protein may be a potential target in cervical cancer therapeutics.

## Materials and methods

### Human tissue samples

In total, 55 normal cervical tissue samples were collected from patients who underwent hysterectomy for benign gynecological diseases and 70 cervical cancer tissue samples were collected from patients who underwent colposcopy biopsy from September 2015 to September 2021 at Women’s Hospital, Zhejiang University School of Medicine, China. Informed consents were obtained from patients for sample collection, and the study was approved by the Hospital Ethical Committee. Detailed information was shown in Table S1.

### Cell culture and treatment

The human cervical cancer cell lines SiHa and C33a were obtained from the American Type Culture Collection (ATCC, USA), CaSki cells were obtained from the Cell Resource Center, Shanghai Institute of Life Sciences, Chinese Academy of Sciences (China), and HeLa and HaCat cells were purchased from the Shanghai Institute of Biochemistry and Cell Biology, Chinese Academy of Sciences (Shanghai, China). SiHa and HaCat cells were cultured in DMEM (BasalMedia, L110KJ), CaSki and C33a cells were cultured in RPMI-1640 (BasalMedia, L210KJ), and HeLa cells were cultured in MEM (BasalMedia, L510KJ) medium containing 10% FBS (Everyday Green, 13011-8611). All cell lines were grown at 37 °C in 5% CO_2_.

### Reagents and antibodies

Reagents utilized in this study included: 3-Methyladenine (3-MA; Millipore Sigma, M9281), bafilomycin A1 (Baf-A1; MedChem Express, HY-100558), Chloroquine (CQ; MedChem Express, HY-17589A), Z-VAD-FMK (Z-VAD; MedChem Express, HY-16658B), Necrostatin-1 (Nec-1; MedChem Express, HY-15760), Ferrostatin-1 (Fer-1; MedChem Express, HY-100579), Liproxstatin-1 (Lip-1; MedChem Express, HY-12726) and R162(MedChem Express, HY-103096). Antibodies used in this study were: LC3B (Milliopore Sigma, L7543), SQSTM1/p62 (Milliopore Sigma, P0067), GAPDH (Diagbio, db106), HuR/ELAVL1 (ABclonal Technology, A19622), GLUD1 (Santa Cruz Biotechnology, sc-515542), AGO2 (ABclonal Technology, A19709) and Flag (MultiSciences, 70-AB002-100).

### RNA extraction, RNase R, and qRT-PCR

Total RNA was extracted from cells or tissues using TRIzol reagent (Invitrogen, 15596018). For RNase R treatment, samples were incubated for 20 min with or without 2 U/μg RNase R at 37 °C (Epicentre Technologies, RNR07250). qRT-PCR analyses were performed using the HiScript III RT SuperMix for qPCR (+gDNA wiper) (Nanjing Vazyme Biotech, R323-01).and ChamQ Universal SYBR qPCR Master Mix (Nanjing Vazyme Biotech, Q711-02). All the primers are presented in Table S2. To validate backspliced junction of circRNA, RNase R digested RNA samples were synthesized into cDNA with random primers. In particular, divergent primers designed at the distal ends of circRNA were used to determine the abundance of circRNA.

### RNA-fluorescence in situ hybridization (FISH) assay

The Kit was purchased from RiboBio (Guangzhou, C10910). The specific probe targeting the back-splice junction of circTICRR was labeled with Cy3, and probe sequences are listed in Table S3. The FISH assay was conducted as previously described [44].

### Transmission electron microscopy (TEM)

TEM was used to observe the autophagosomes and autolysosomes in cervical cancer cell lines. Briefly, cells were first fixed with 2.5% glutaraldehyde in 0.1 M phosphate at 4 °C buffer overnight and postfixed with 1% OsO_4_ for 1–2 h at room temperature. After dehydration and infiltration, embedding, ultrathin sectioning and staining were performed. Finally, the cells were observed in Hitachi Model H-7650 TEM.

### Gene knockdown and overexpression

The small interfering RNAs (siRNAs) targeting circTICRR, HuR, and GLUD1 were all synthesized by GenePharma Biotech (Shanghai, China). The sequences of all oligonucleotides are listed in Table S4. Expression plasmids for circTICRR were designed and synthesized by Geneseed Biotech (Guangdong, China), and GLUD1 was constructed by Weizhen Biotech (Shandong, China). Moreover, 3xFlag-tagged HuR protein, HuR RRM domain-truncated plasmids and HuR mutation plasmid were synthesized by Genechem Biotech (Shanghai, China). Transient transfection of the siRNAs and the overexpression plasmids were performed using Lipofectamine RNAiMAX Reagent (Invitrogen, 13778150) or X-tremeGENE™ HP DNA Transfection Reagent (Roche, 06366546001) according to the manufacturer’s instructions.

### CCK-8 cell growth assay

Cell growth was detected by Cell Counting Kit 8 assays (Dojindo, CK04).

### Cell apoptosis analysis

Cell apoptosis assay was analyzed in the SiHa and CaSki cells by Flow Cytometry using ANXA5/Annexin V-FITC and propidium iodide (PI) Kit (Multisiences, 70-APCC101-100) according to the manufacturer’s instructions. And ANXA5/Annexin V -APC and 7-AAD Kit (Multisiences, China, 70-AP105-100) was employed in HeLa cells and cells treated with peptides. Apoptosis in tissues of animal models was carried out by routine Terminal deoxynucleotidyl transferase dUTP nick-end Labeling (TUNEL) method (Servicebio, G1507), where the apoptosis index = (apoptotic cell/total cell)*100%.

### Autophagic flux measurement

mRFP-GFP-LC3 (Hanbio Biotechnology, HB-AP210 0001) was utilized to monitor autophagic flux. Reagent was added at an MOI = 20 in SiHa cells, 100 in CaSki cells, and 10 in HeLa cells for 24 h according to the manufacturer’s instructions followed by siRNAs or plasmids for another 48 h. Finally, fluorescence images were captured using Olympus microscope.

### Western blot

Cellular proteins were extracted using cell lysis buffer (Solarbio, R0010) and 1:100 Phenylmethylsulfonyl fluoride (PMSF; Solarbio, P0100). Proteins were separated by 4-20% SurePAGE gels (GenScript, m00657) and transferred to PVDF membranes (Bio-Rad, 1620177). The membranes were exposed to ImageQuant LAS 4000 mini (ImageQuant LAS 4000 mini, USA).

### Northern blot

The junction probe for circTICRR was synthesized and labeled with digoxigenin (DIG, shown in Table S3). The protocol was performed as previously described [26].

### RNA sequencing assay

Total RNA of SiHa cells (1 × 10^7^) transfected with si-NC (*n* = 3) or si-circTICRR#2 (*n* = 3) was extracted in accordance with the manual of TRIzol® reagent (Invitrogen, Carlsbad, CA, USA). The RNA-seq and analysis were carried out on the illumina PE150 at RiboBioCo, Ltd. (Guangzhou, China).

### RNA pull-down

Biotin-labeled RNA probes were synthesized by TsingKe (China) (shown in Table S3). Then RNA pull-down assays were executed using a Pierce™ Magnetic RNA-Protein Pull-down Kit (Thermo Scientific, 20164). Briefly, streptavidin magnetic beads were incubated with circTICRR or GLUD1 sense probes and antisense probes with agitation for 30 min at room temperature. After two washes, the cell lysates were incubated with beads in RNA-Protein binding buffer at 4 °C overnight. The proteins attached to the magnetic beads were eluted after washing three times using wash buffer for western blot analysis or subsequent mass spectrometry (MS) analysis (Lumingbio, Shanghai, China).

### RNA immunoprecipitation (RIP)

The RIP assay was conducted using an EZ-Magna RIP kit (Millipore, 17-704) according to the manufacturer’s instructions. Briefly, protein A/G magnetic beads were incubated with antibodies for 30 min at room temperature. After two washes with RIP wash buffer, cell lysates were incubated with beads at 4 °C overnight. Co-precipitated RNA was extracted and detected by qRT-PCR.

### Nude mouse xenograft experiment

Animal experiments were approved by the Animal Ethical and Welfare Committee of Zhejiang Chinese Medical University (granted number: IACUC-20210614-09). There-to five-week-old female BALB/c nude mice were purchased from Shanghai Silaike Laboratory Animal Company, Ltd. (China). Cervical cancer xenografts were established by injecting SiHa or CaSki cells (1 × 10^7 cells of 100μl PBS) into the left armpit of the mice. One week after cell inoculation, si-circTICRR#2 or si-NC was intratumorally injected at a concentration of 75ρmol/20μl. The tumor volume was measured once a week for 4 weeks, and the volume was calculated according to the following formula: Tumor volume (mm^3^) = (length×width^2^)/2.

### Interfering peptide synthesis and usage

The interfering peptides blocking the interaction between circTICRR and HuR were synthesized by ChinaPeptides (Shanghai, China). The interfering peptides were synthesized by linking the biotin-labeled cell-penetrating peptide (YGRKKRRQRRR) at the N-terminus and FITC at the C-terminus with the core amino acids of HuR (purity>95%). All the sequences of peptides are listed in Table S5. For cell treatment, peptides (30 μmol/l) were applied to SiHa and CaSki cells for further uptake observation, RNA pull-down, CCK-8 assays, apoptosis analysis, and western blot analysis. For in vivo experiments, peptides (3 mg/kg) were intratumorally injected every three days.

### Biotin-labeled peptide pull-down

Biotin-labeled peptides (30 μmol/l) were incubated with extracted total RNA at 4 °C overnight. Then, the RNA-peptide complex was incubated with streptavidin magnetic beads (Thermo Scientific, 88817) at 4 °C for 2 h. After four washes, RNAs attached to the beads were extracted and measured by qRT-PCR.

### Statistical analysis

All statistics were analyzed with GraphPad Prism 8.0 (GraphPad Software, USA). Data were examined as the mean ± standard deviation (SD), and Student’s *t* tests were used to analyze the data with normal distribution between two groups. Otherwise, Mann–Whitney tests were used. ANOVA test was used for more than 2 groups. All assays were repeated at least three times. A *p* value < 0.05 was regarded as statistical significance.

## Supplementary information

supplementary materials

checklist

Original Data File

## Data Availability

The datasets used or analyzed during the current study are available from the corresponding author on reasonable request.
