# Supplementary material for: Oncogenic circTICRR suppresses autophagy via binding to HuR protein and stabilizing GLUD1 mRNA in cervical cancer
Source: Cell Death Dis. 2022 May 20;13(5):479. doi: 10.1038/s41419-022-04943-1 (PMC9122915; doi:10.1038/s41419-022-04943-1)
Supplement: Supplementary file 1 — supplementary materials [file 41419_2022_4943_MOESM1_ESM.docx]

**Supplemental materials**

**Oncogenic circTICRR suppresses autophagy via binding to HuR protein and stabilizing GLUD1 mRNA in cervical cancer**

Tingjia Zhu^2*^, Yixuan Cen^2*^, Zhuoye Chen^2^, Yanan Zhang^2^, Lu Zhao^2^, Jiaying Wang^5^, Weiguo Lu^1,2,3^, Xing Xie^1,2^ and Xinyu Wang^1,2,3,4#^

**Table S1.** Information of all specimens

| Case number | pathological diagnosis | 2^−ΔΔCt^ value for circTICRR |  |
| --- | --- | --- | --- |
| N1 | Normal | 1.695879 |  |
| N2 | Normal | 2.865328 |  |
| N3 | Normal | 0.961038 |  |
| N4 | Normal | 1.307067 |  |
| N5 | Normal | 2.964935 |  |
| N6 | Normal | 3.128203 |  |
| N7 | Normal | 0.350949 |  |
| N8 | Normal | 1.14532 |  |
| N9 | Normal | 0.224806 |  |
| N10 | Normal | 6.950338 |  |
| N11 | Normal | 0.558805 |  |
| N12 | Normal | 2.570708 |  |
| N13 | Normal | 0.388998 |  |
| N14 | Normal | 0.765072 |  |
| N15 | Normal | 0.769059 |  |
| N16 | Normal | 1.30029 |  |
| N17 | Normal | 7.532204 |  |
| N18 | Normal | 0.029712 |  |
| N19 | Normal | 1.822813 |  |
| N20 | Normal | 0.548603 |  |
| N21 | Normal | 0.648095 |  |
| N22 | Normal | 1.459976 |  |
| N23 | Normal | 1.056855 |  |
| N24 | Normal | 2.671594 |  |
| N25 | Normal | 0.673256 |  |
| N26 | Normal | 1.01521 |  |
| N27 | Normal | 0.22978 |  |
| N28 | Normal | 1.282553 |  |
| N29 | Normal | 1.087781 |  |
| N30 | Normal | 0.561916 |  |
| N31 | Normal | 1.702804 |  |
| N32 | Normal | 1.184462 |  |
| N33 | Normal | 0.290992 |  |
| N34 | Normal | 1.363882 |  |
| N35 | Normal | 1.56373 |  |
| N36 | Normal | 0.347009 |  |
| N37 | Normal | 1.536685 |  |
| N38 | Normal | 4.269681 |  |
| N39 | Normal | 1.918743 |  |
| N40 | Normal | 0.428259 |  |
| N41 | Normal | 2.090728 |  |
| N42 | Normal | 0.523292 |  |
| N43 | Normal | 1.182325 |  |
| N44 | Normal | 1.512522 |  |
| N45 | Normal | 0.4035 |  |
| N46 | Normal | 0.288367 |  |
| N47 | Normal | 2.891761 |  |
| N48 | Normal | 0.469334 |  |
| N49 | Normal | 0.053949 |  |
| N50 | Normal | 1.646685 |  |
| N51 | Normal | 2.296316 |  |
| N52 | Normal | 3.200365 |  |
| N53 | Normal | 3.34043 |  |
| N54 | Normal | 1.025534 |  |
| N55 | Normal | 0.952666 |  |
| SCC1 | cervical squamous cell carcinoma | 0.311722 | |
| SCC2 | cervical squamous cell carcinoma | 1.035743 | |
| SCC3 | cervical squamous cell carcinoma | 3.937226 | |
| SCC4 | cervical squamous cell carcinoma | 1.362416 | |
| SCC5 | cervical squamous cell carcinoma | 2.003238 | |
| SCC6 | cervical squamous cell carcinoma | 3.925533 | |
| SCC7 | cervical squamous cell carcinoma | 5.552827 | |
| SCC8 | cervical squamous cell carcinoma | 1.947335 | |
| SCC9 | cervical squamous cell carcinoma | 10.26425 | |
| SCC10 | cervical squamous cell carcinoma | 1.989248 | |
| SCC11 | cervical squamous cell carcinoma | 0.43 | |
| SCC12 | cervical squamous cell carcinoma | 3.246509 | |
| SCC13 | cervical squamous cell carcinoma | 0.96 | |
| SCC14 | cervical squamous cell carcinoma | 4.871016 | |
| SCC15 | cervical squamous cell carcinoma | 3.845931 | |
| SCC16 | cervical squamous cell carcinoma | 6.371153 | |
| SCC17 | cervical squamous cell carcinoma | 1.246986 | |
| SCC18 | cervical squamous cell carcinoma | 6.143711 | |
| SCC19 | cervical squamous cell carcinoma | 7.636381 | |
| SCC20 | cervical squamous cell carcinoma | 9.871938 | |
| SCC21 | cervical squamous cell carcinoma | 6.733006 | |
| SCC22 | cervical squamous cell carcinoma | 3.01 | |
| SCC23 | cervical squamous cell carcinoma | 2.135801 | |
| SCC24 | cervical squamous cell carcinoma | 0.983807 | |
| SCC25 | cervical squamous cell carcinoma | 7.647142 | |
| SCC26 | cervical squamous cell carcinoma | 2.150019 | |
| SCC27 | cervical squamous cell carcinoma | 2.113649 | |
| SCC28 | cervical squamous cell carcinoma | 0.667042 | |
| SCC29 | cervical squamous cell carcinoma | 1.14744 | |
| SCC30 | cervical squamous cell carcinoma | 2.313906 | |
| SCC31 | cervical squamous cell carcinoma | 0.820846 | |
| SCC32 | cervical squamous cell carcinoma | 3.782198 | |
| SCC33 | cervical squamous cell carcinoma | 9.116956 | |
| SCC34 | cervical squamous cell carcinoma | 0.197797 | |
| SCC35 | cervical squamous cell carcinoma | 1.640511 | |
| SCC36 | cervical squamous cell carcinoma | 3.487925 | |
| SCC37 | cervical squamous cell carcinoma | 0.612737 | |
| SCC38 | cervical squamous cell carcinoma | 0.69771 | |
| SCC39 | cervical squamous cell carcinoma | 1.608876 | |
| SCC40 | cervical squamous cell carcinoma | 1.999054 | |
| SCC41 | cervical squamous cell carcinoma | 1.187765 | |
| SCC42 | cervical squamous cell carcinoma | 1.352378 | |
| SCC43 | cervical squamous cell carcinoma | 1.115114 | |
| SCC44 | cervical squamous cell carcinoma | 0.399562 | |
| SCC45 | cervical squamous cell carcinoma | 0.160485 | |
| SCC46 | cervical squamous cell carcinoma | 0.765286 | |
| SCC47 | cervical squamous cell carcinoma | 0.464938 | |
| SCC48 | cervical squamous cell carcinoma | 0.499525 | |
| SCC49 | cervical squamous cell carcinoma | 1.418233 | |
| SCC50 | cervical squamous cell carcinoma | 0.866685 | |
| SCC51 | cervical squamous cell carcinoma | 1.191387 | |
| SCC52 | cervical squamous cell carcinoma | 0.570199 | |
| SCC53 | cervical squamous cell carcinoma | 1.384278 | |
| SCC54 | cervical squamous cell carcinoma | 0.836959 | |
| SCC55 | cervical squamous cell carcinoma | 2.781804 | |
| SCC56 | cervical squamous cell carcinoma | 0.785105 | |
| SCC57 | cervical squamous cell carcinoma | 0.940284 | |
| SCC58 | cervical squamous cell carcinoma | 3.070531 | |
| SCC59 | cervical squamous cell carcinoma | 0.390195 | |
| SCC60 | cervical squamous cell carcinoma | 3.099921 | |
| SCC61 | cervical squamous cell carcinoma | 0.564719 | |
| SCC62 | cervical squamous cell carcinoma | 0.519815 | |
| SCC63 | cervical squamous cell carcinoma | 1.14744 | |
| SCC64 | cervical squamous cell carcinoma | 4.87699 | |
| SCC65 | cervical squamous cell carcinoma | 2.009836 | |
| SCC66 | cervical squamous cell carcinoma | 2.728998 | |
| SCC67 | cervical squamous cell carcinoma | 5.742282 | |
| SCC68 | cervical squamous cell carcinoma | 1.456712 | |
| SCC69 | cervical squamous cell carcinoma | 0.57873 | |
| SCC70 | cervical squamous cell carcinoma | 2.592912 |  |

**Table S2.** Sequences of primers used for qRT-PCR in this study

| Item | Sequence | |
| --- | --- | --- |
| circTICRR | Forward (5’-3’) | AGGCCCAGAAGTTACATCCAGA |
|  | Reverse (5’-3’) | TCGGCAAGGCAATGTATTAATTCGC |
| circTICRR  (full length) | Forward (5’-3’) | ATGAATACCATGTGCCGTTCCTTAA |
|  | Reverse (5’-3’) | CTTCTGTCTCACTGGAGTACGAGG |
| linearTICRR | Forward (5’-3’) | GAATGTCGCAAGGCTGAATGT |
|  | Reverse (5’-3’) | AGTGTTCTTCCTCTGTCTTCCA |
| 18S | Forward (5’-3’) | TTAATTCCGATAACGAACGAGA |
|  | Reverse (5’-3’) | CGCTGAGCCAGTCAGTGTAG |
| GAPDH | Forward (5’-3’) | TCACCACCATGGAGAAGGC |
|  | Reverse (5’-3’) | GCTAAGCAGTTGGTGGTGCA |
| U1 | Forward (5’-3’) | CCATGATCACGAAGGTGGTTT |
|  | Reverse (5’-3’) | ATGCAGTCGAGTTTCCCACAT |
| HuR | Forward (5’-3’) | AAGCACCCGAAGACGGTTAG |
|  | Reverse (5’-3’) | GCTGCGAAAAGCACATGGAA |
| GLUD1 | Forward (5’-3’) | CCATTGTACCCACGGCAGAG |
|  | Reverse (5’-3’) | GCCAGAGTGCACGATGTCTT |

**Table S3.** Sequences of probes used in this study

| Item | Sequence |
| --- | --- |
| circTICRR FISH probe | 5’Cy3-GCAGCCTGTAGTAACCTTTAG- 3’Cy3 |
| 18S FISH probe | 5’Cy3-CATTCGCAGTTTCACTGTACCGGCC- 3’Cy3 |
| U6 FISH probe | 5’Cy3-GAACGCTTCACGAATTTGCGTGTCATCCTTGCGCA- 3’Cy3 |
| circTICRR pull-down  sense probe | Biotin-GCAGCCTGTAGTAACCTTTAG |
| circTICRR pull-down antisense probe | Biotin-CTAAAGGTTACTACAGGCTGC |
| GLUD1mRNA pull-down sense probe | Biotin- TGACGAGGCATTATCACACG |
| GLUD1mRNA pull-down antisense probe | Biotin- CGTGTGATAATGCCTCGTCA |
| circTICRR Northern blot probe | GATGTGGCTGGGGAGAAAGGAATCCAAAAGATACCTAGTGGGAGAACAGTGGATAAATTGGAAGACAGAGGAAGAACACTAAGAAGTTCTAAACCTAAAGGTTACTACAGGCTGCCTCAGCTAATAAGGAAGAGTCTTCCAAAACTGAAGGCGAATTAATACATTGCCTTGCCGAGCTCTACCAGAGAAAATCTCGTGAAGAATCCACTATAGCTCATCAAGAAGACAGCAAAAAGAAACG |

**Table S4.** Sequences of siRNAs against specific targets in this study

| Item | Sequence | |
| --- | --- | --- |
| circTICRR siRNA-1 | Sense (5’-3’) | CUAAACCUAAAGGUUACUATT |
|  | Antisense (5’-3’) | UAGUAACCUUUAGGUUUAGTT |
| circTICRR siRNA-2 | Sense (5’-3’) | CUAAAGGUUACUACAGGCUTT |
|  | Antisense (5’-3’) | AGCCUGUAGUAACCUUUAGTT |
| GLUD1  siRNA-1 | Sense (5’-3’) | CCCAAGAACUAUACUGAUATT |
|  | Antisense (5’-3’) | UAUCAGUAUAGUUCUUGGGTT |
| GLUD1 siRNA-2 | Sense (5’-3’) | GCGUUCUGCCAGGCAAAUUTT |
|  | Antisense (5’-3’) | AAUUUGCCUGGCAGAACGCTT |
| HuR  siRNA-1 | Sense (5’-3’) | GAGGCAAUUACCAGUUUCATT |
|  | Antisense (5’-3’) | UGAAACUGGUAAUUGCCUCTT |
| HuR  siRNA-2 | Sense (5’-3’) | GCGACUUCAACACCAACAATT |
|  | Antisense (5’-3’) | UUGUUGGUGUUGAAGUCGCTT |
| Ctrl siRNA | Sense (5’-3’) | UUCUCCGAACGUGUCACGUTT |
|  | Antisense (5’-3’) | ACGUGACACGUUCGGAGAATT |

**Table S5.** Sequences of peptide in this study

| Peptides | Sequences |
| --- | --- |
| CTL-1 | YGRKKRRQRRR-GGFVAHSKGNVYLK |
| HIP-1 | YGRKKRRQRRR-VAGHSLGYGFVNK |
| CTL-2 | YGRKKRRQRRR-CIILGNSGFQAWDYK |
| HIP-2 | YGRKKRRQRRR-SGWCIFIYNLGQDAK |
| CTL-3 | YGRKKRRQRRR-FFMGTNNGTCKVTKK |
| HIP-3 | YGRKKRRQRRR-TNKCKGFGFVTMTNK |


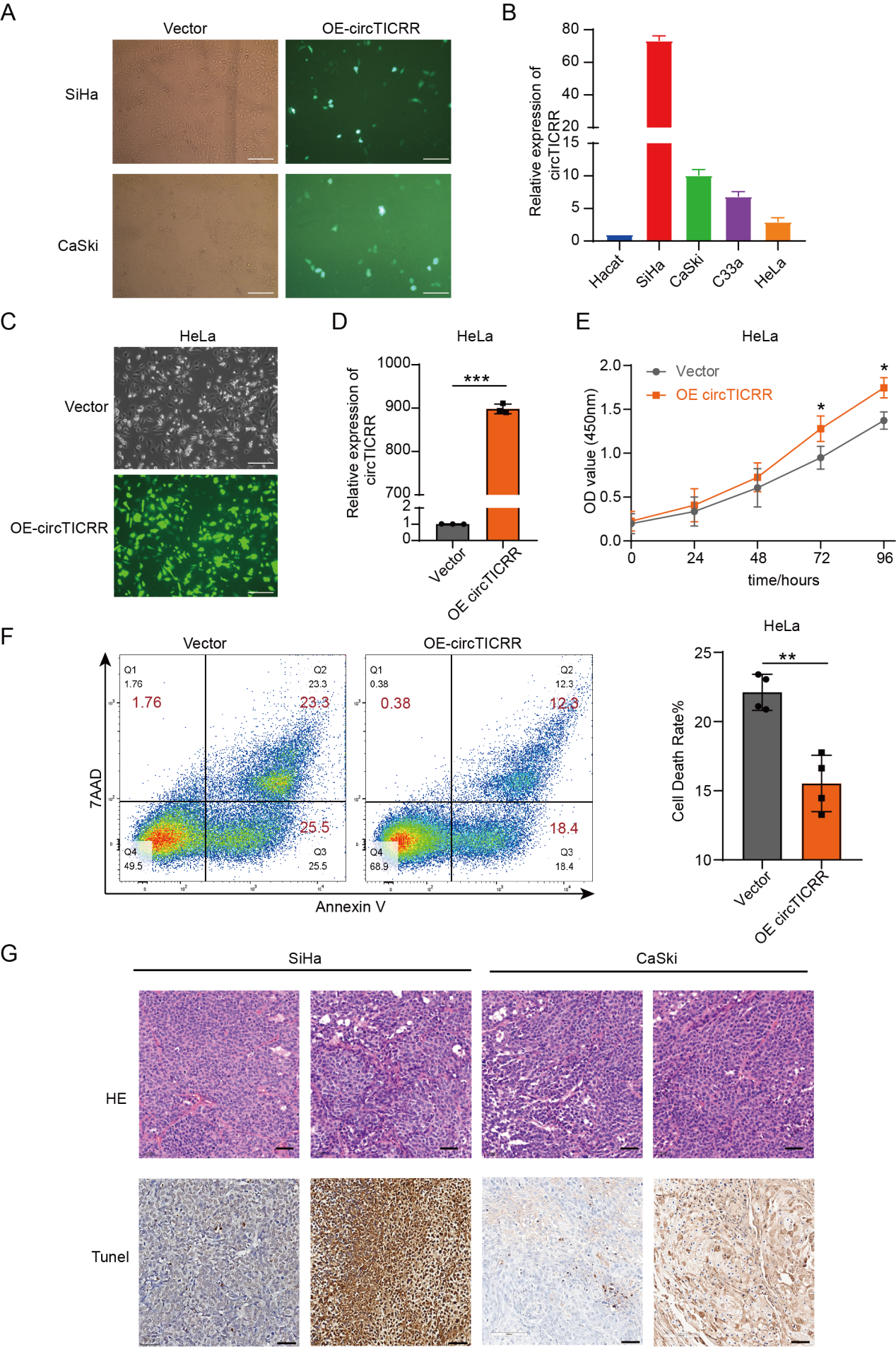


**Figure S1. related to Figure 2.**

(**A**)**.** SiHa and CaSki cells were transfected with circTICRR overexpression plasmids with GFP fluorescent protein for 48 h. Then transfection efficiency was determined by the green signal through fluorescent microscopy. Scar bar: 200 μm.

(**B**)**.** The relative circTICRR levels were detected by qRT-PCR in 4 cervical cancer cell lines and negative control cell line Hacat.

(**C**)**.** The transfection efficiency in the HeLa cells with circTICRR overexpression plasmid for 48 h was determined through fluorescent microscope. Scar bar: 200 μm.

(**D**)**.** The relative circTICRR level was detected by qRT-PCR in the HeLa cells with circTICRR overexpression plasmid or control vector.

(**E**)**.** The proliferation in the HeLa cells with circTICRR overexpression plasmid or control vector was detected CCK-8 assay.

(**F**)**.** Apoptosis in the HeLa cells with circTICRR overexpression plasmid or control vector was measured by Flow Cytometry.

(**G**)**.**Images of hematoxylin and eosin (H&E) and apoptotic cells detected by TUNEL assay in xenografts treated with circTICRR siRNA#2 or negative control siRNA. Scale bar, 50 μm.

Data are representative of at least three independent experiments and presented as the mean ± SD. **p* < 0.05, ***p* < 0.01.


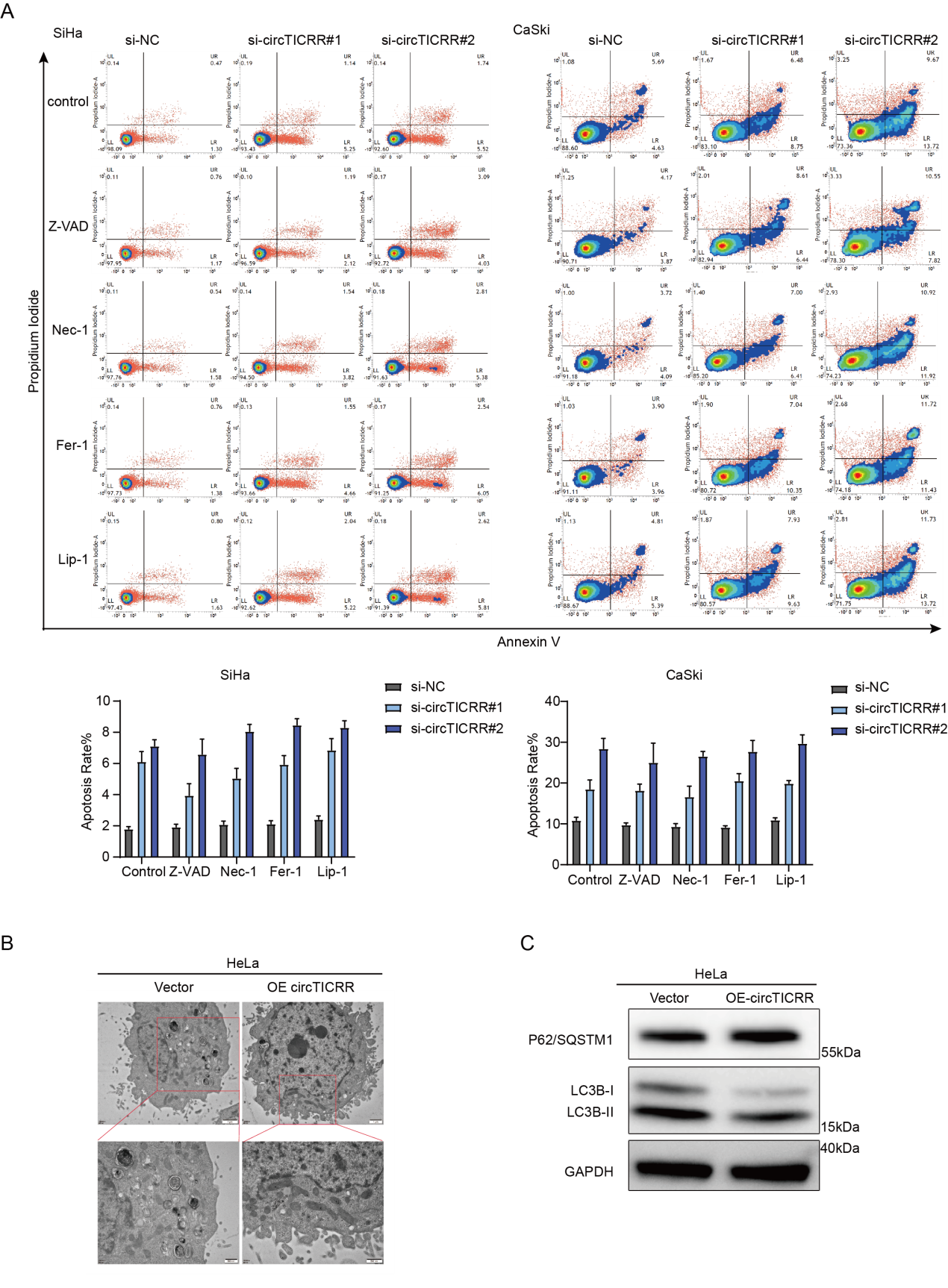


**Figure S2. related to Figure 3.**

(**A**)**.** SiHa and CaSki cells were pretreated with Z-VAD (10 μM), Nec-1 (10 μM), Fer-1 (2 μM), Lip-1 (2 μM), and normal medium, respectively, for 1 h, followed by combined treatment with two circTICRR siRNAs for additional 48 h. Cell apoptosis was measured by Flow Cytometry.

(**B-C**)**.** Cell morphology (B) and autophagy-related proteins (C) were monitored by TEM and western blot, respectively.

Data are representative of at least three independent experiments and presented as the mean ± SD.


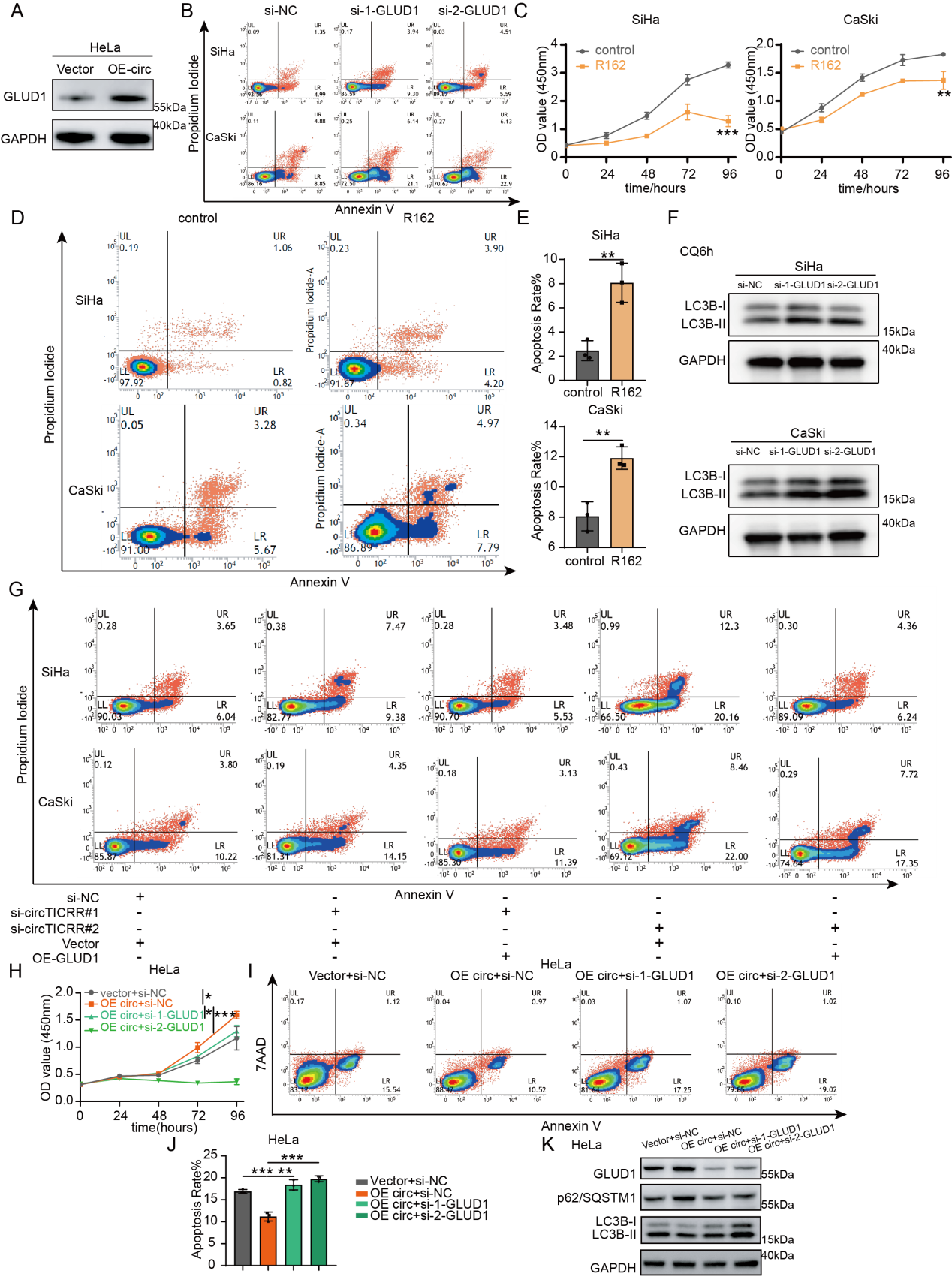


**Figure S3. related to Figure 4.**

(**A**)**.** The levels of GLUD1 protein expression detected by western blot in the HeLa cells with circTICRR overexpression or control vector.

(**B**)**.** Apoptosis was measured by Flow Cytometry in the SiHa and CaSki cells with GLUD1 knockdown.

(**C-E**)**.** The proliferation detected by CCK-8 assay (**C**) and apoptosis detected by Flow Cytometry (**D** and **E**) in the SiHa (40 μM) and CaSki (100 μM) cells treated with GLUD1 inhibitor R162.

(**F**)**.** Autophagy-related protein LC3B-I/II was monitored by western blot in the SiHa and CaSki cells transfected with two GLUD1 siRNAs or a negative control for 72 h followed by Autophagy inhibitor CQ treatment for another 6 h.

(**G**)**.** Apoptosis was detected by Flow Cytometry in the SiHa and CaSki cells transfected with si-circTICRR#1, si-circTICRR#2, si-circTICRR#1 plus GLUD1 plasmid, si-circTICRR#2 plus GLUD1 plasmid, and negative control, respectively.

(**H-K**)**.** The proliferation detected by CCK-8 assay (**H**), apoptosis detected by Flow Cytometry (**I** and **J**),and autophagy-related proteins detected by western blot (**K**) in the HeLa cells with circTICRR plasmid, circTICRR plasmid plus si-GLUD1#1, circTICRR plasmid plus si-GLUD1#2, and negative control, respectively.

Data are representative of at least three independent experiments and presented as the mean ± SD. **p* < 0.05, ***p* < 0.01, ****p* < 0.001.
