# Supplementary material for: Oncogenic circTICRR suppresses autophagy via binding to HuR protein and stabilizing GLUD1 mRNA in cervical cancer
Source: Cell Death Dis. 2022 May 20;13(5):479. doi: 10.1038/s41419-022-04943-1 (PMC9122915; doi:10.1038/s41419-022-04943-1)

Figure 3c

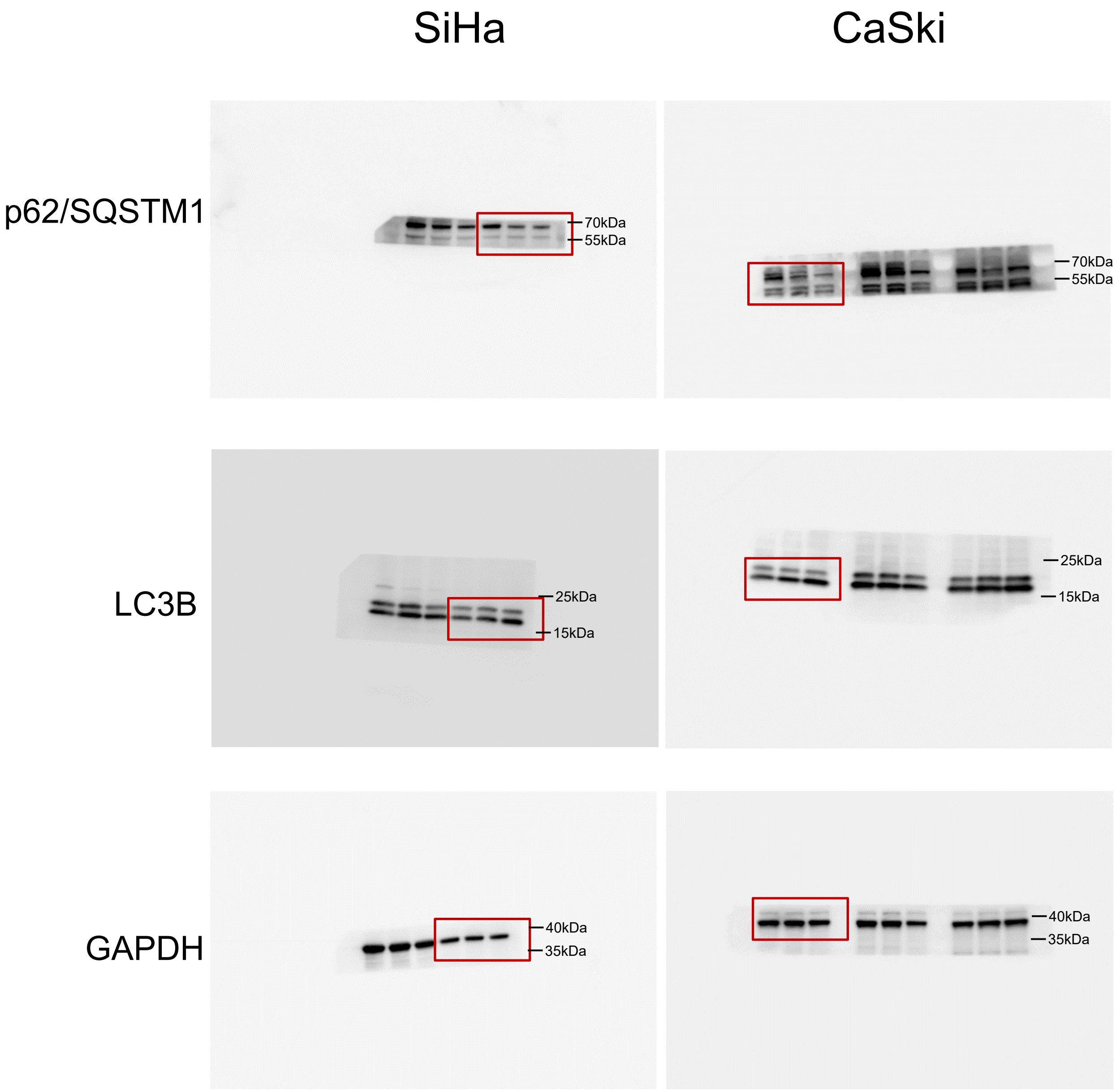
SiHa
CaSki
p62/SQSTM1
70kDa
55kDa
70kDa
55kDa
LC3B
25kDa
15kDa
25kDa
15kDa
GAPDH
40kDa
35kDa
40kDa
35kDa

Figure 3d

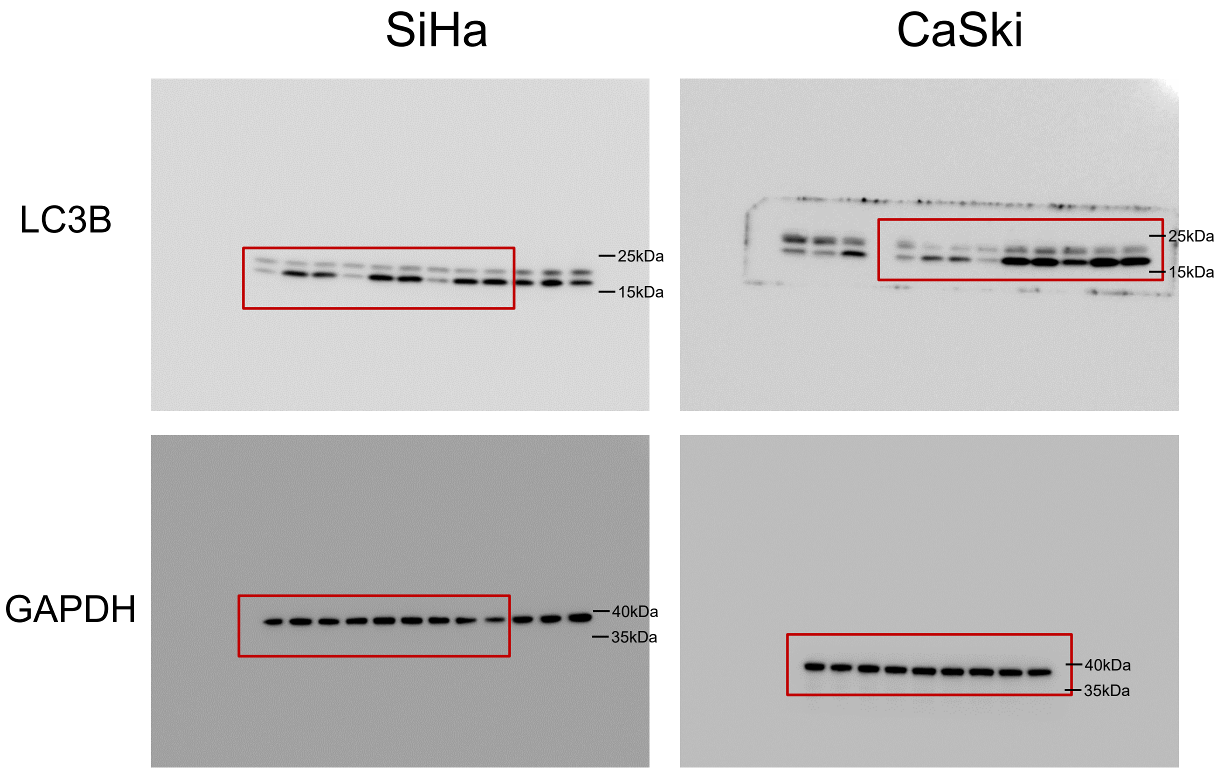
SiHa
CaSki
LC3B
GAPDH
25kDa
15kDa
25kDa
15kDa
40kDa
35kDa
40kDa
35kDa

Figure 4b

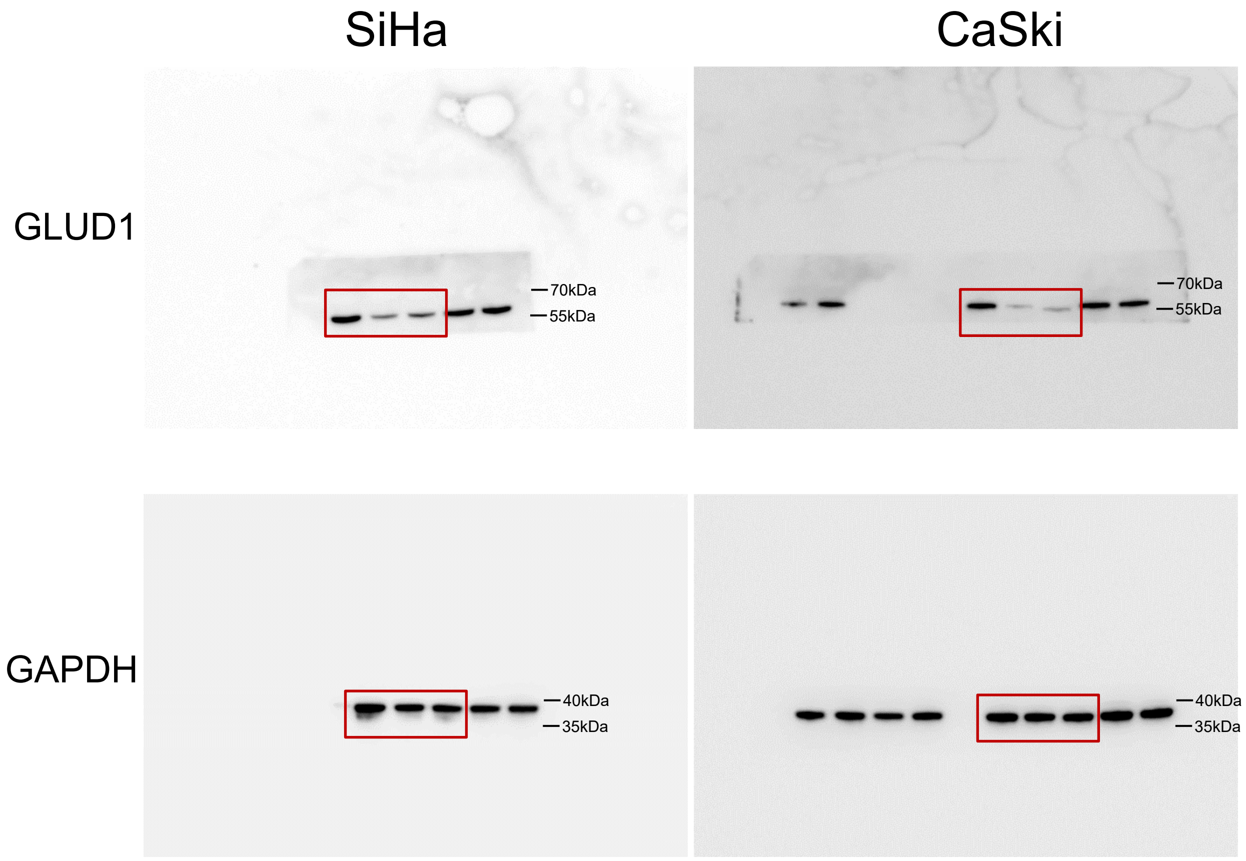

SiHa
CaSki
GLUD1
GAPDH
70kDa
55kDa
70kDa
55kDa
40kDa
35kDa
40kDa
35kDa

Figure 4f

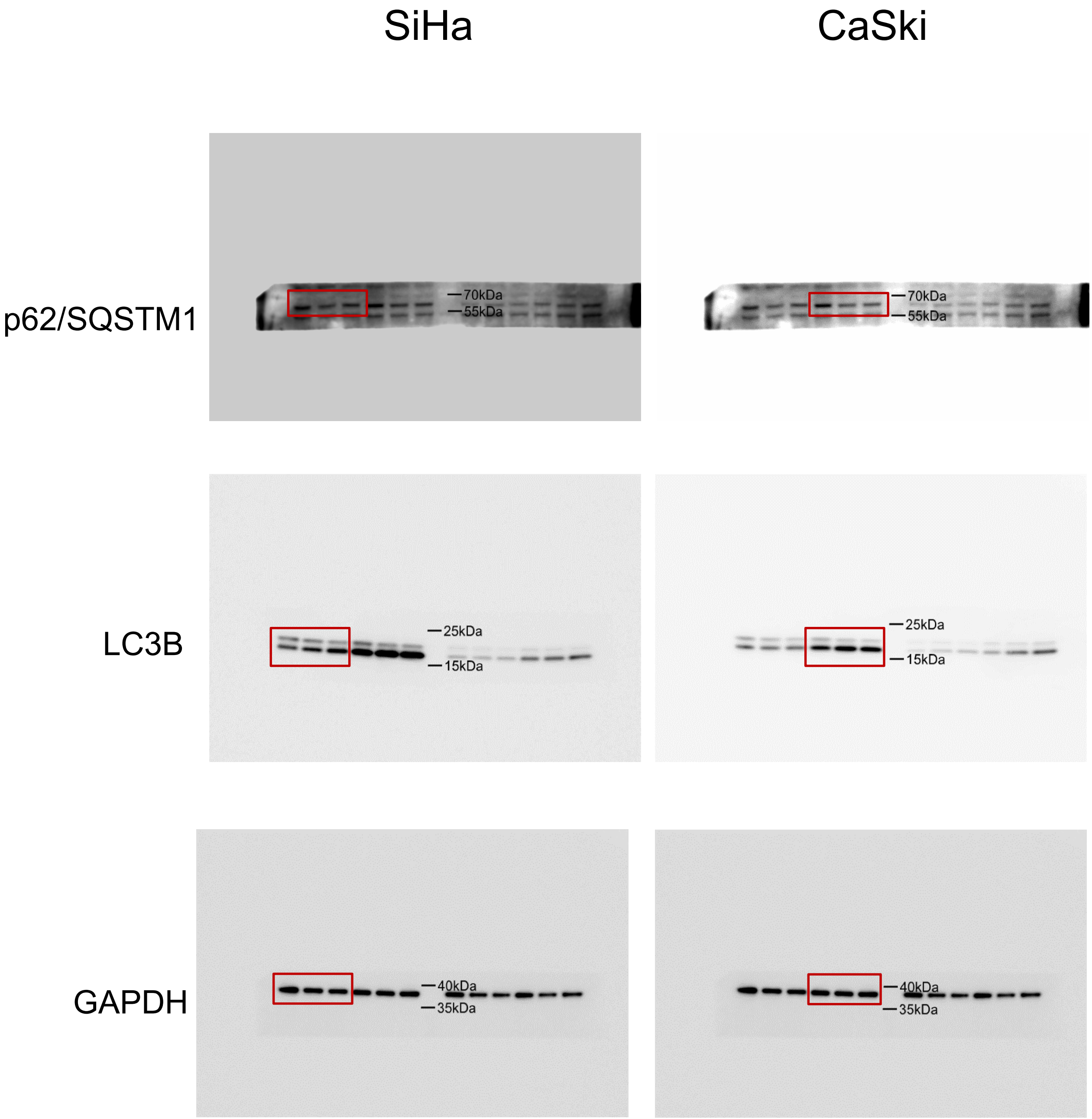

SiHa
CaSki
p62/SQSTM1
70kDa
55kDa
70kDa
55kDa
LC3B
25kDa
15kDa
25kDa
15kDa
GAPDH
40kDa
35kDa
40kDa
35kDa

Figure 4j

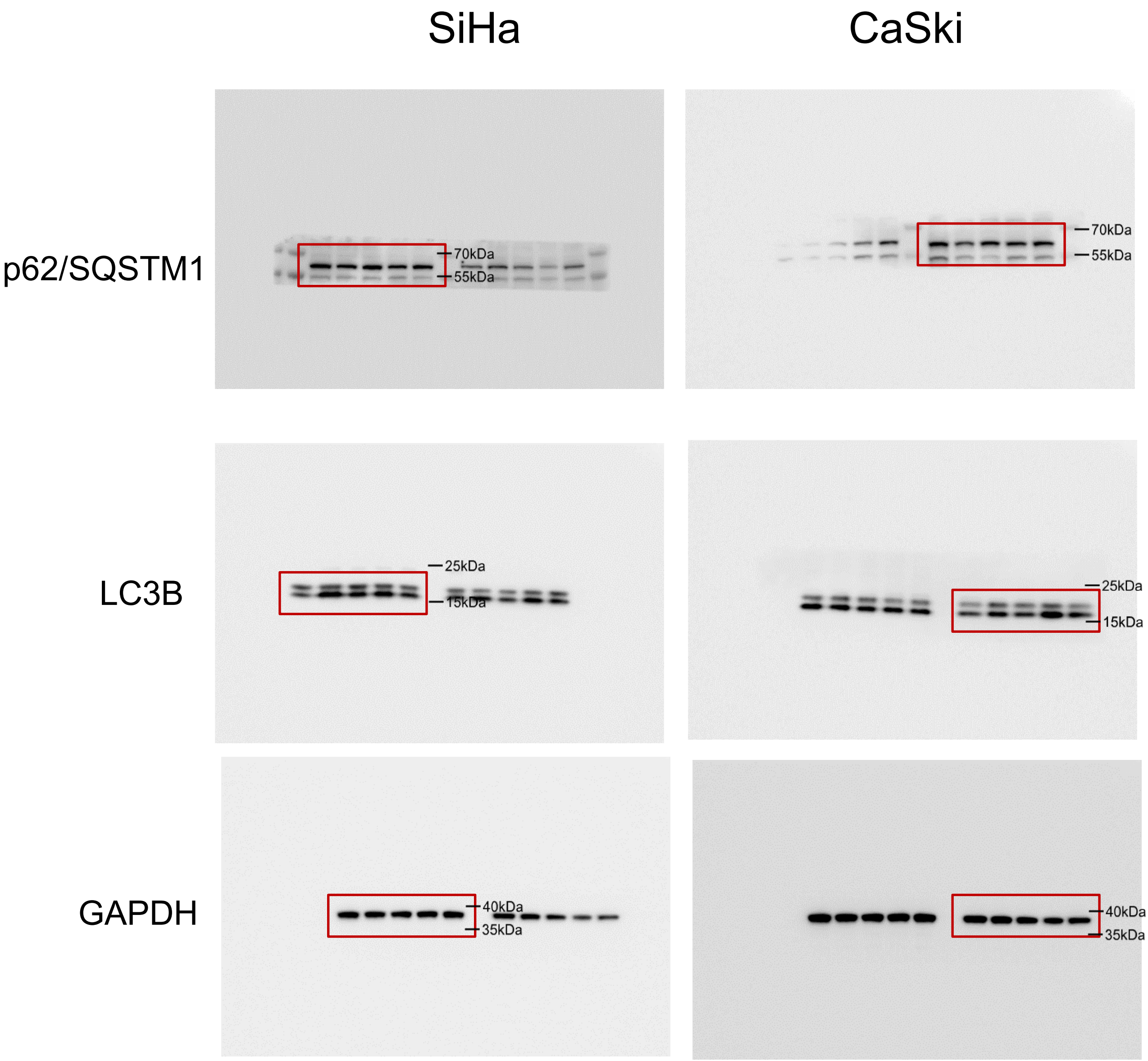

SiHa
CaSki
p62/SQSTM1
70kDa
55kDa
70kDa
55kDa
LC3B
25kDa
15kDa
25kDa
15kDa
GAPDH
40kDa
35kDa
40kDa
35kDa

Figure 5c

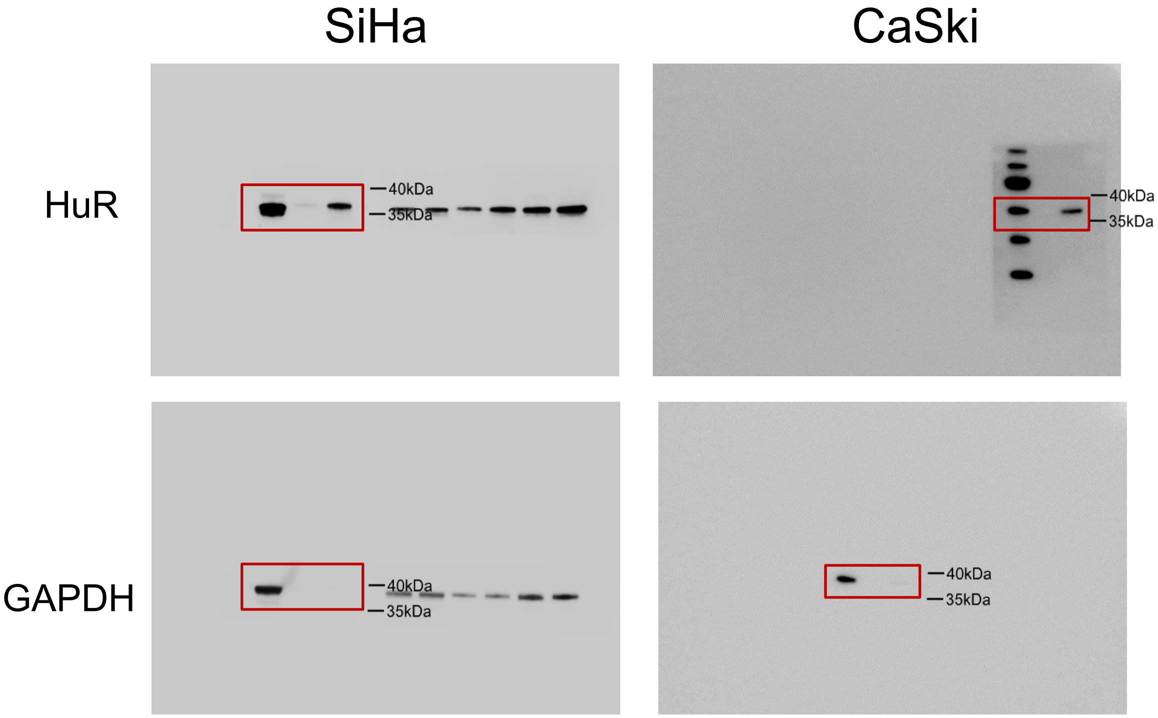

Figure 5e

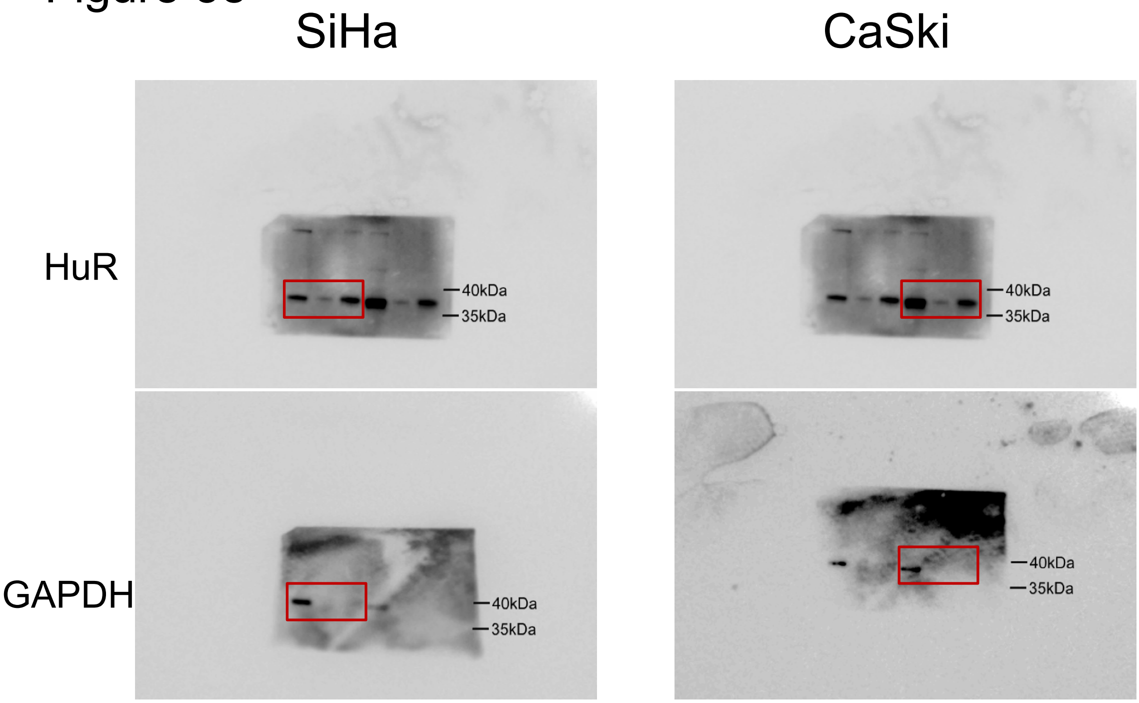

Figure 5h

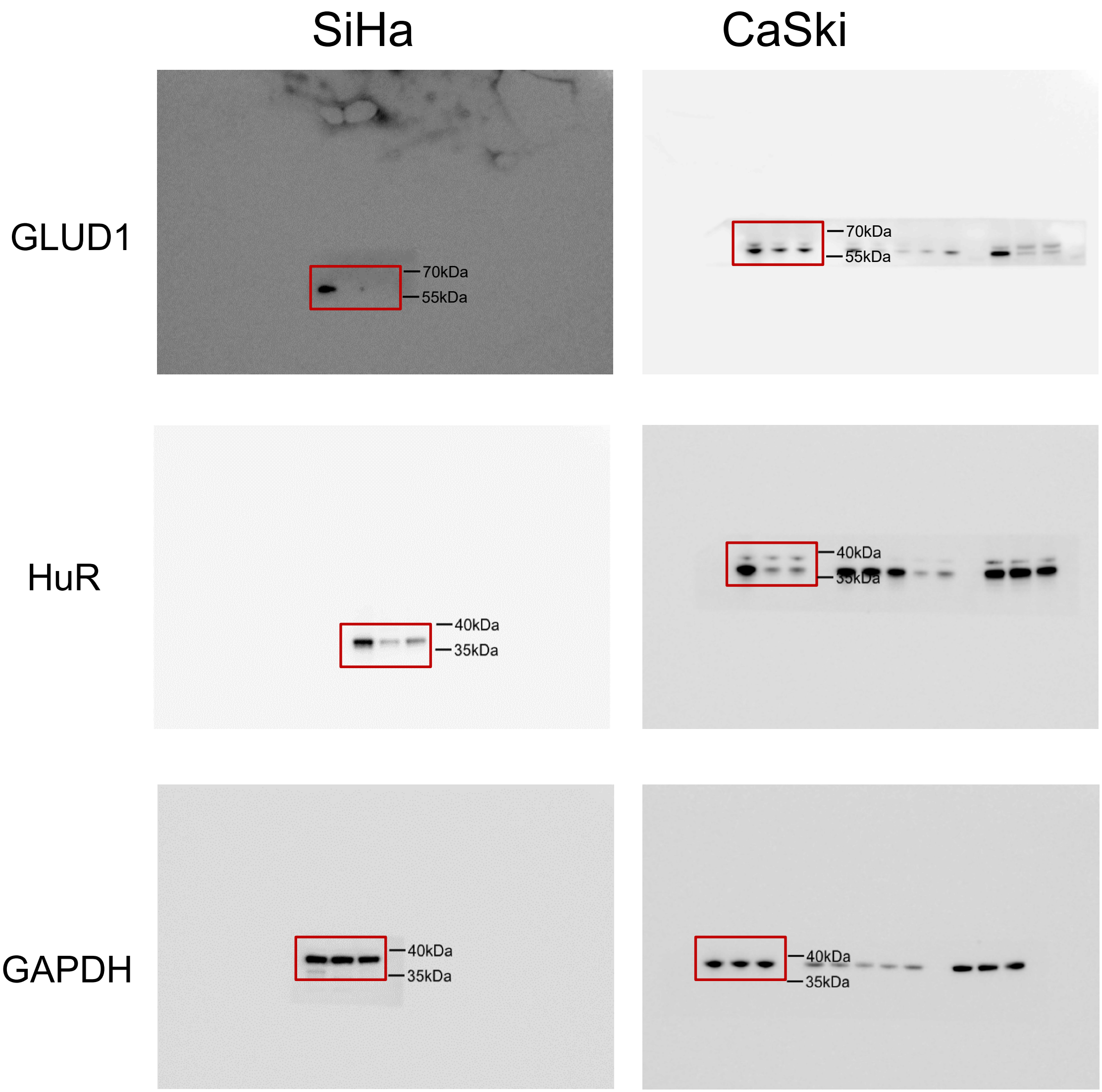
SiHa
CaSki
GLUD1
70kDa
55kDa
70kDa
55kDa
HuR
40kDa
35kDa
40kDa
35kDa
GAPDH
40kDa
35kDa
40kDa
35kDa

Figure 5k

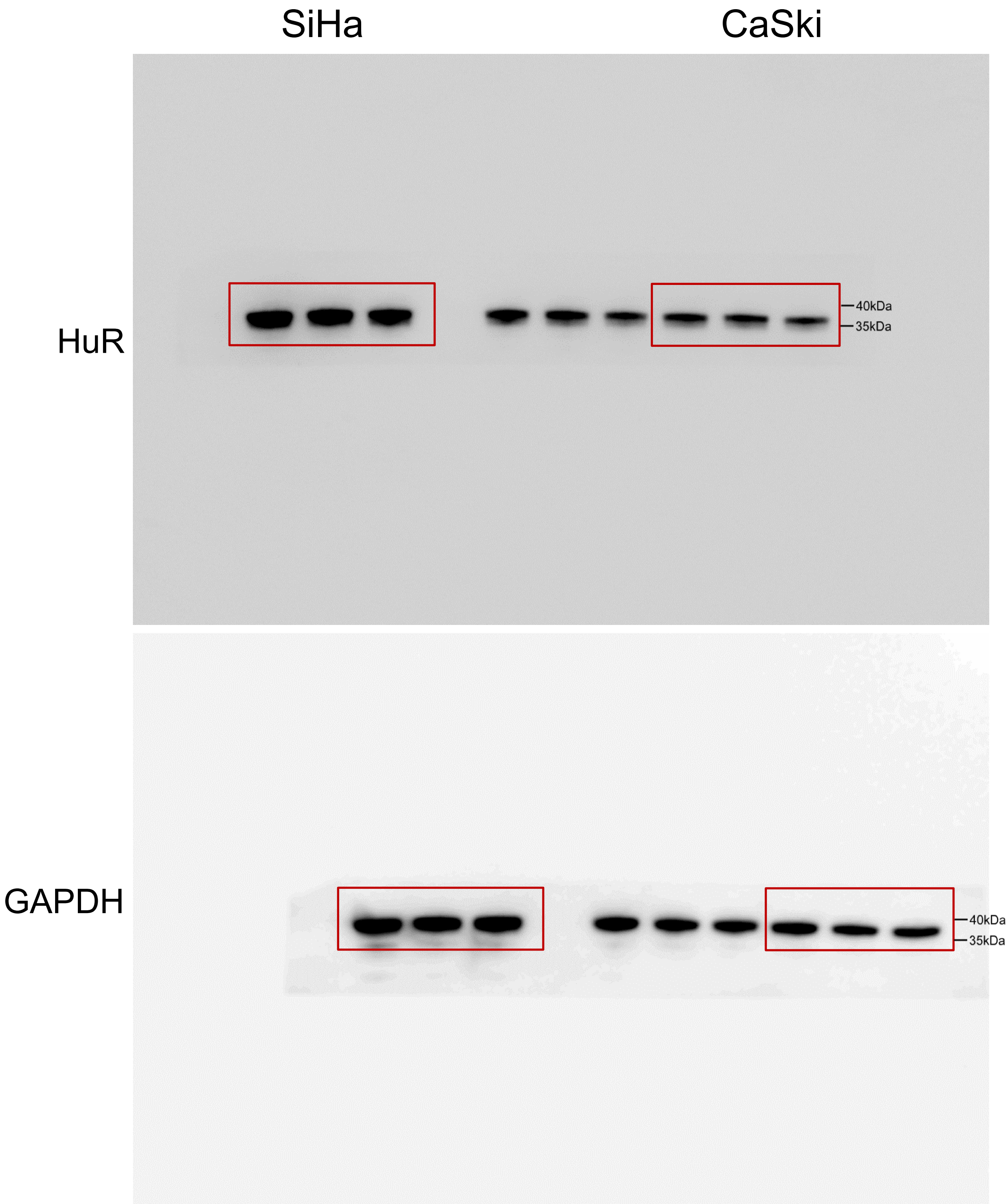

Figure 6b

HeLa

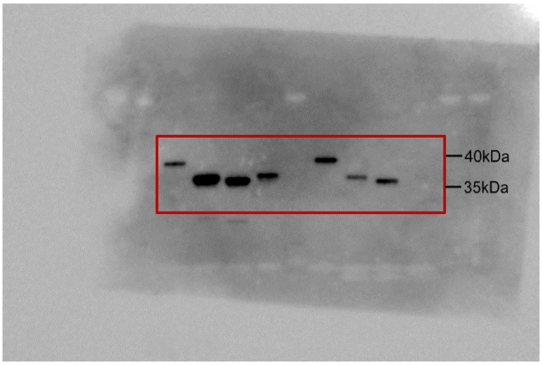

Flag

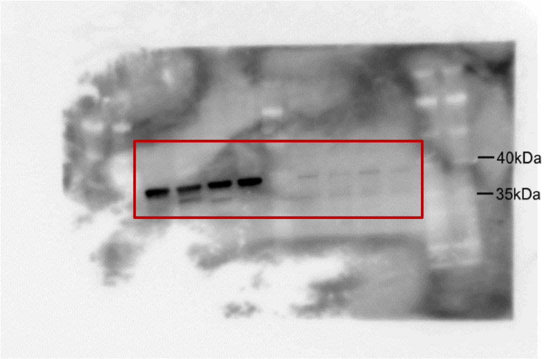

GAPDH

Figure 6e

HeLa

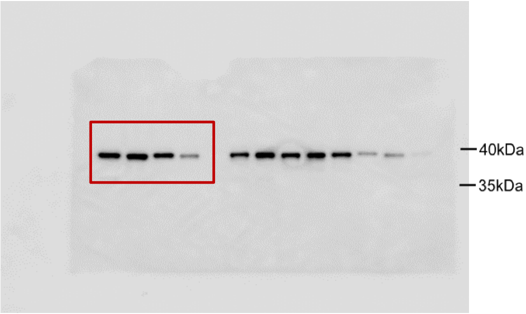

Flag-HuR

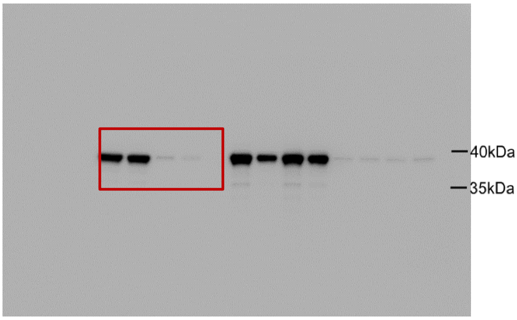

GAPDH

Figure 6g

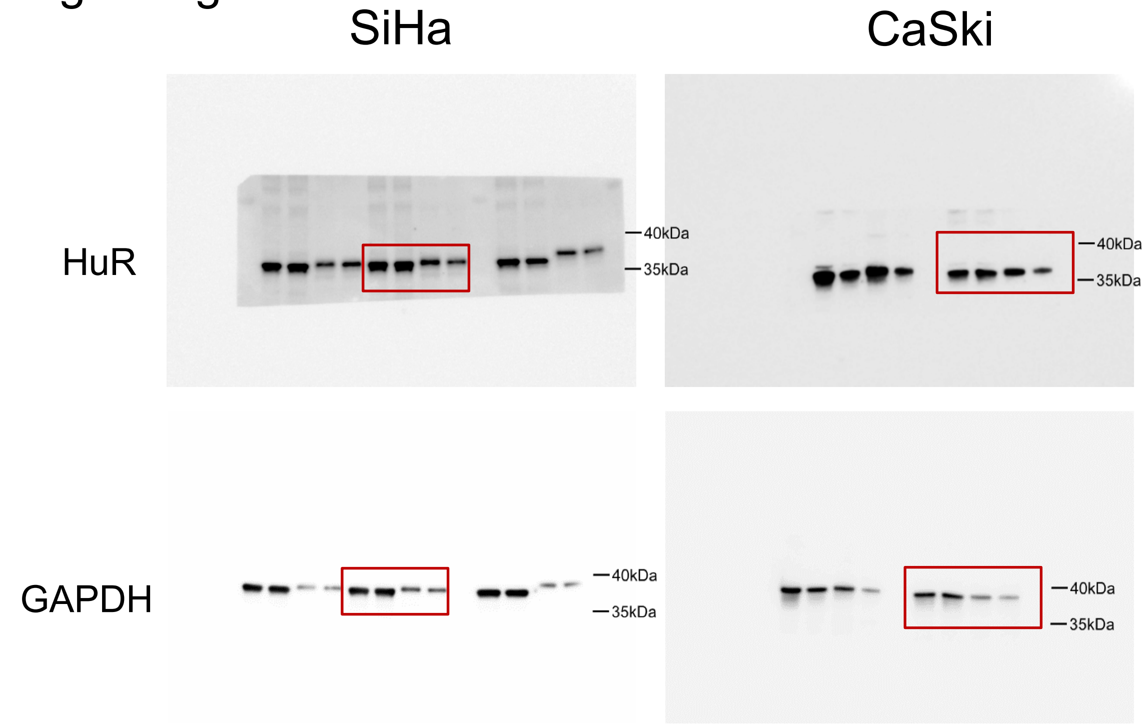

Figure 6j

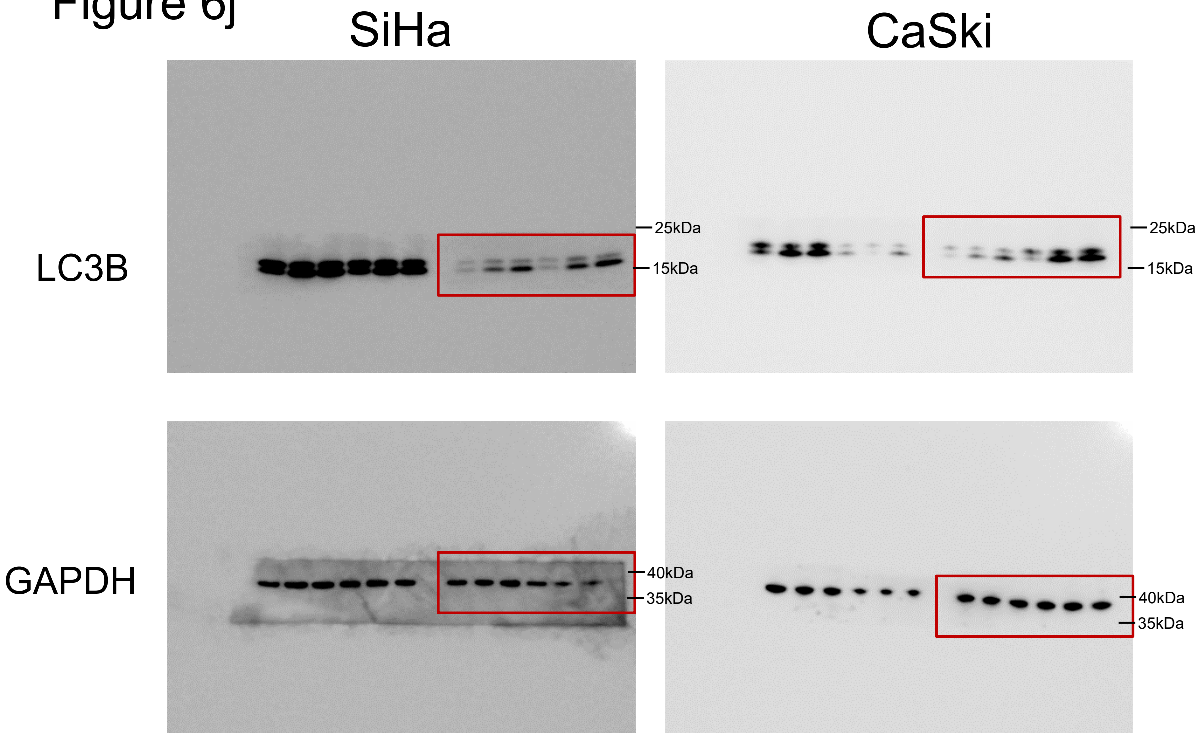

Figure S2c

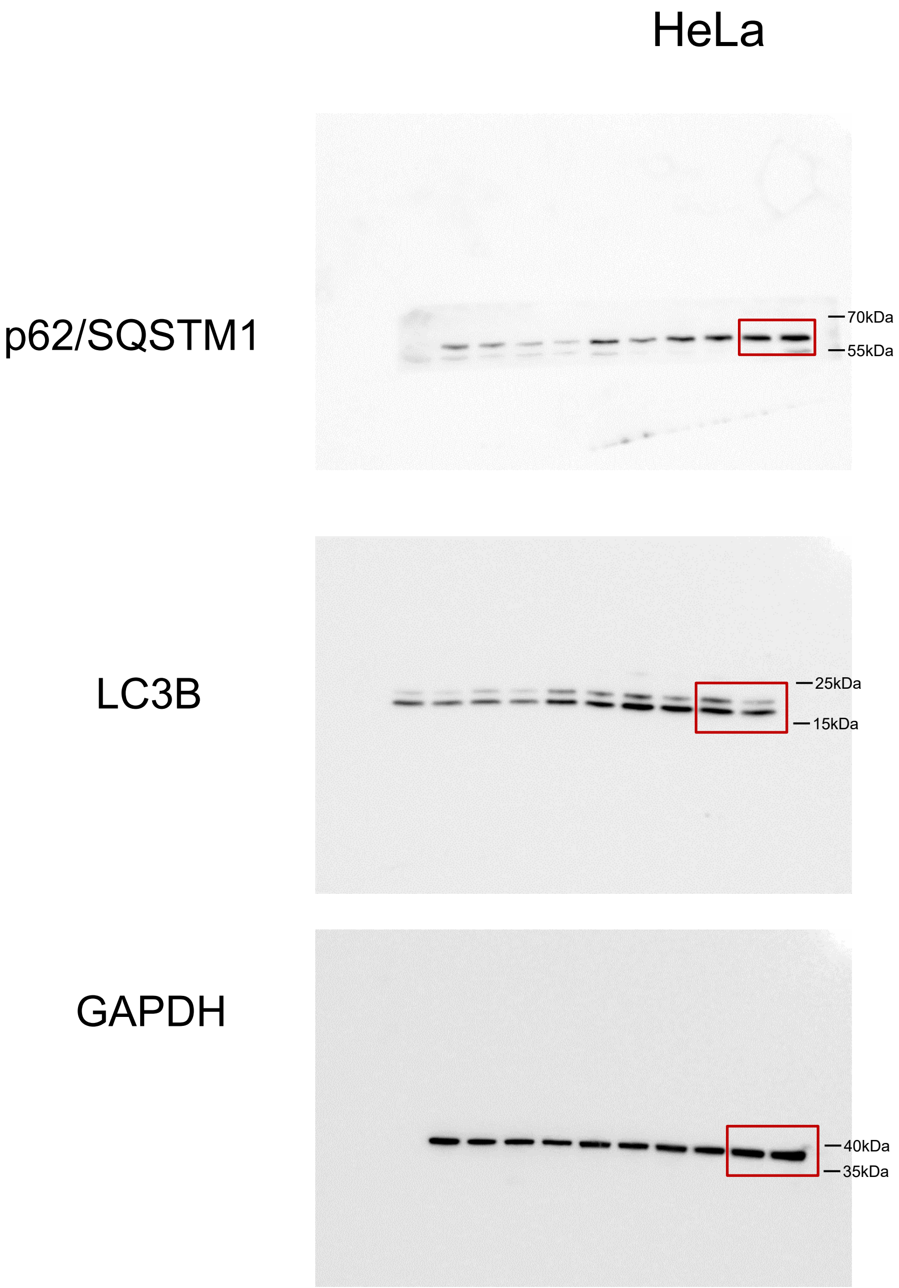
HeLa
p62/SQSTM1
70kDa
55kDa
LC3B
25kDa
15kDa
GAPDH
40kDa
35kDa

Figure S3a

HeLa

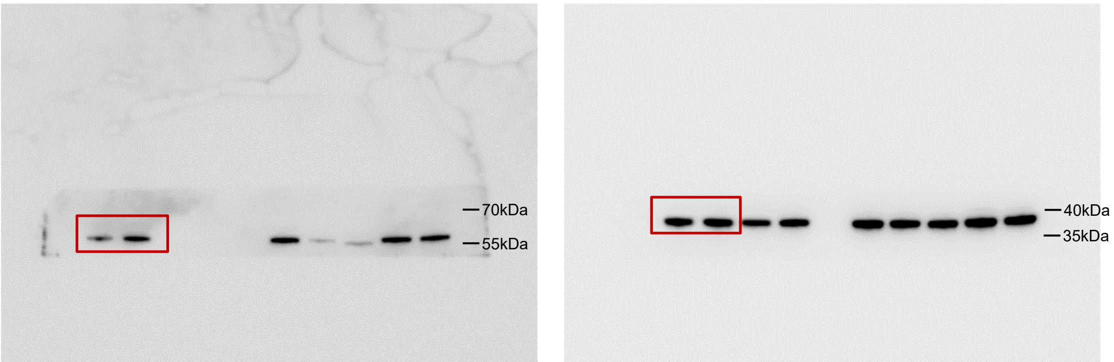

GLUD1 GAPDH

Figure S3f

SiHa CaSki

LC3B

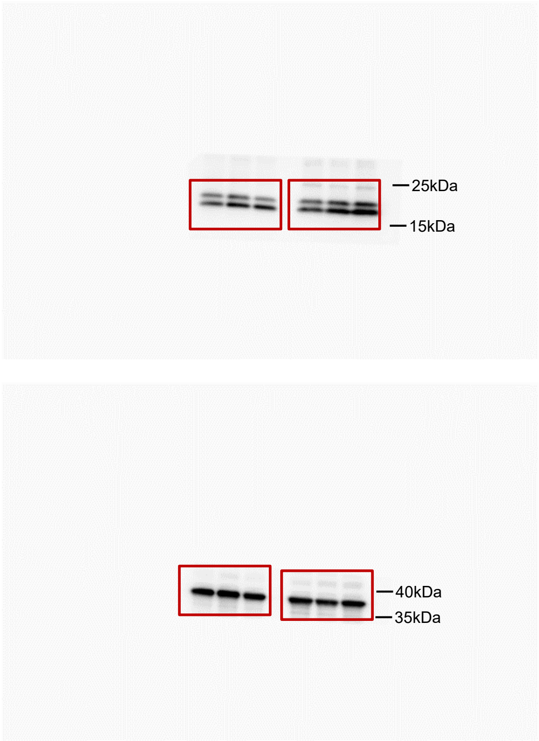

GAPDH

Figure S3k

HeLa

GLUD1

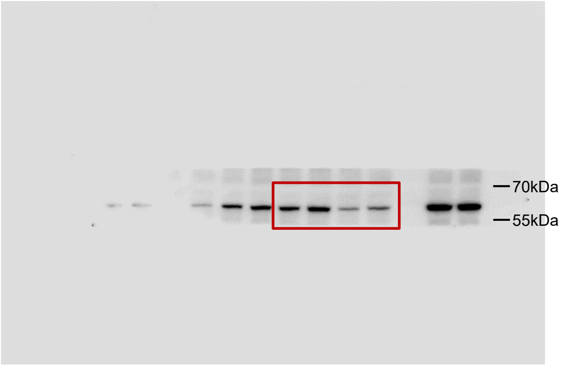

P62

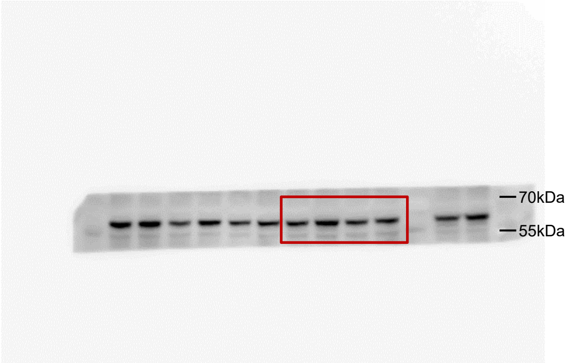

LC3B

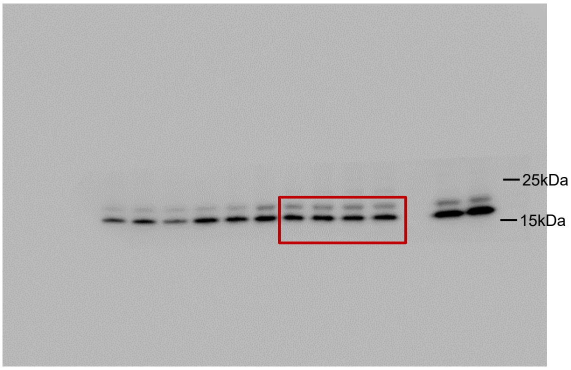

GAPDH

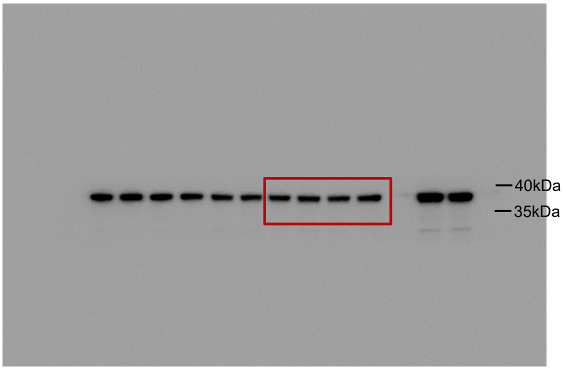

Supplement: Supplementary file 3 — Original Data File [file 41419_2022_4943_MOESM3_ESM.pdf]
